# 5-HTTLPR–environment interplay and its effects on neural reactivity in adolescents

**DOI:** 10.1016/j.neuroimage.2012.07.067

**Published:** 2012-11-15

**Authors:** Nicholas D. Walsh, Tim Dalgleish, Valerie J. Dunn, Rosemary Abbott, Michelle C. St Clair, Matthew Owens, Graeme Fairchild, William S. Kerslake, Lucy V. Hiscox, Luca Passamonti, Michael Ewbank, Maria Ban, Andrew J. Calder, Ian M. Goodyer

**Affiliations:** a Developmental and Life-course Research Group, Department of Psychiatry, University of Cambridge, Cambridge, CB2 8AD, UK; b Medical Research Council Cognition and Brain Sciences Unit, Cambridge CB2 7EF, UK; c School of Psychology, University of Southampton, Southampton, SO17 1BJ, UK; d Consiglio Nazionale delle Ricerche (CNR), Istituto di Scienze Neurologiche (ISN), Unità di Ricerca Neuroimmagini, 88100, Catanzaro, Italy; e Department of Clinical Neurosciences, University of Cambridge, Addenbrooke's Hospital, CB2 0SP, Cambridge, UK

**Keywords:** 5-HTTLPR, (serotonin-transporter-linked polymorphic region), SLC6A4, (Solute carrier family 6 (neurotransmitter transporter, serotonin), member 4), CA, (Childhood adversity), PH, (Psychiatric history), RNLE, (Recent negative life events), CAMEEI, (Cambridge Early Experience Interview), fMRI, (functional magnetic resonance imaging), MFQ, (Mood and Feelings Questionnaire), SAI, (Spielberger Anxiety Inventory), K-SADS-PL, (Kiddie Schedule for Affective Disorders and Schizophrenia for School-Age Children—Present and Lifetime version, 5-HTTLPR, Childhood adversity, Recent negative life events, Amygdala, Functional magnetic resonance imaging, Faces

## Abstract

It is not known how 5-HTTLPR genotype × childhood adversity (CA) interactions that are associated with an increased risk for affective disorders in population studies operate at the neural systems level. We hypothesized that healthy adolescents at increased genetic and environmental risk for developing mood disorders (depression and anxiety) would demonstrate increased amygdala reactivity to emotional stimuli compared to those with only one such risk factor or those with none. Participants (n = 67) were classified into one of 4 groups dependent on being homozygous for the long or short alleles within the serotonin-transporter-linked polymorphic region (5-HTTLPR) of the SLC6A4 gene and exposure to CA in the first 11 years of life (present or absent). A functional magnetic resonance imaging investigation was undertaken which involved viewing emotionally-salient face stimuli. In addition, we assessed the role of other variables hypothesized to influence amygdala reactivity, namely recent negative life-events (RNLE) assessed at ages 14 and 17, current anxiety symptoms and psychiatric history. We replicated prior findings demonstrating moderation by gene variants in 5-HTTLPR, but found no support for an effect of CA on amygdala reactivity. We also found a significant effect of RNLE aged 17 with amygdala reactivity demonstrating additive, but not interactive effects with 5-HTTLPR. A whole-brain analysis found a 5-HTTLPR × CA interaction in the lingual gyrus whereby CA appears to differentially modify neural reactivity depending on genotype. These results demonstrate that two different forms of environmental adversities interplay with 5-HTTLPR and thereby differentially impact amygdala and cortical reactivity.

## Highlights

► Tested hypothesis that amygdala is neural locus for 5-HTTLPR × environment interaction ► Additive effects of 5-HTTLPR genotype and recent life events on amygdala reactivity ► Genotype × childhood adversity interaction on neural reactivity in lingual gyrus ► Two forms of environmental adversities interplay with 5-HTTLPR and neural reactivity. ► No support for hypothesized G × E interaction on amygdala reactivity

## Introduction

The amygdala is a neural region fundamental for emotion processing (Adolphs et al., 2005; Tye et al., 2011). Heightened amygdala reactivity to emotionally-salient stimuli assessed using functional neuroimaging, is a putative neurocognitive marker of elevated genetic and/or environmental risk for affective disorder (Domschke and Dannlowski, 2010; Scharinger et al., 2010). The aim of the present study is to clarify the role of hypothesized genetic and environmental risk factors that may moderate amygdala reactivity.

The most studied genetic moderator of amygdala reactivity has been allelic variation in the serotonin-transporter-linked-promoter region (5-HTTLPR) of the *SLC6A4* gene (Alexander et al., 2012; Bertolino et al., 2005; Brown and Hariri, 2006; Canli et al., 2005, 2006; Dannlowski et al., 2008, 2010; Drabant et al., 2012; Friedel et al., 2009; Furman et al., 2011; Furmark et al., 2004, 2009; Gillihan et al., 2010; Hariri et al., 2002, 2005; Heinz et al., 2005, 2007; Klucken et al., 2012; Kobiella et al., 2011; Lau et al., 2009; Lemogne et al., 2011; Lonsdorf et al., 2011; Schardt et al., 2010; Smolka et al., 2007; von dem Hagen et al., 2011; Wolfensberger et al., 2008). Through meta-analysis, it has been demonstrated that individuals carrying the less efficient short allele of the 5-HTTLPR, the gene variant associated with increased susceptibility for affective disorders, reliably demonstrate increased amygdala reactivity when exposed to negative or arousing environmental stimuli (Munafo et al., 2008; Murphy et al., 2012).

Similarly, it has been claimed that amygdala reactivity is moderated by environmental stressors known to increase risk for affective psychopathology. When the effects of the adverse environment are investigated in population and clinical studies it is important to measure and elucidate the differential effects arising from distal, proximate and current negative experiences. This comprehensive measurement of the social environment to determine their relative contributions to a defined outcome has yet to be applied to neuroimaging studies designed to reveal individual differences in amygdala reactivity. Prior brain imaging studies of the effects distal stressors such as childhood adversity (CA) on amygdala reactivity have tended neither to determine nor control for effects arising from recent or current stressors (Canli et al., 2006; Chugani et al., 2001; Dannlowski et al., 2012; Grant et al., 2011; Hsu et al., 2010; Lemogne et al., 2011; Maheu et al., 2010; McCrory et al., 2011; Taylor et al., 2006; Tottenham et al., 2011; Williams et al., 2009) though some attempts have been made in this regard (van Harmelen et al., 2012).

As well as the importance of recent negative life events (Admon et al., 2009; Beblo et al., 2006); current stress or negative emotional state (Barrett et al., 2007; Bishop et al., 2004), negative personality traits (Calder et al., 2011; Etkin et al., 2004), and current or past psychiatric disorder (Etkin and Wager, 2007; Fu et al., 2004; Victor et al., 2010) are potential confounds when attempting to determine the effects of genetic or distal environmental experiences upon amygdala reactivity. Indeed, as the above participant factors are strongly associated with prior CA (Kendler et al., 2002), it is necessary to partition and experimentally control for these in order to unambiguously elucidate the role of CA upon amygdala reactivity.

Studies in healthy adults screened for current or prior psychiatric history, that reported a significant association between 5-HTTLPR variation and amygdala reactivity, attempted to eliminate the influence of psychiatric history when isolating the genetic effect (Alexander et al., 2012; Bertolino et al., 2005; Brown and Hariri, 2006; Canli et al., 2005, 2006; Dannlowski et al., 2010; Drabant et al., 2012; Gillihan et al., 2010; Gillihan et al., 2011; Hariri et al., 2002, 2005; Heinz et al., 2005, 2007; Klucken et al., 2012; Lemogne et al., 2011; Lonsdorf et al., 2011; Rao et al., 2007; Roiser et al., 2009; Schardt et al., 2010; Smolka et al., 2007; von dem Hagen et al., 2011). However, less is known of the role of distal and proximate negative life events and current negative emotional state upon amygdala reactivity in prior 5-HTTLPR studies [though see (Dannlowski et al., 2010; Gillihan et al., 2010, 2011; Lemogne et al., 2011; Rao et al., 2007) which did include current mood and life-event covariates in their analyses].

There is thus a clear need to elucidate the influence of CA, either as a main effect and/or in combination with 5-HTTLPR genotype, on amygdala reactivity while also taking account the putative effects of these aforementioned other variables. The optimal design would include careful assessment of recent negative life events (RNLE), current mood, and psychiatric history (PH) in a group free of current psychiatric illness, of known genetic profile, and for whom valid data concerning CA have been acquired.

By selecting individuals from the ROOTS cohort, a longitudinal study of adolescent development (Dunn et al., 2011; Goodyer et al., 2010), we were able to identify participants who carried either the l/l or s/s 5-HTTLPR variants and who had either experienced childhood adversities (CA+) or not (CA−) in the first 11 years of life. In addition we were able to examine the role of distal childhood adverse experiences whilst controlling for more proximate and concurrent variables hypothesized to influence amygdala reactivity, namely recent negative life-events (RNLE) collected when participants were aged 14 and 17, current anxiety symptoms and psychiatric history. Using functional magnetic resonance imaging (fMRI) and an emotional face-processing task we undertook a precise investigation of the other factors likely to influence amygdala reactivity described above. To that end, our design incorporated a number of methodological advances compared to previous studies (Alexander et al., 2012; Canli et al., 2006; Drabant et al., 2012; Klucken et al., 2012; Lemogne et al., 2011; Maheu et al., 2010; Tottenham et al., 2011; van Harmelen et al., 2012; Williams et al., 2009). The first is that we used well-characterized and well-matched (in terms of age, gender, IQ and socio-economic status) sub-samples of healthy adolescents ascertained from the community. Second we undertook a researcher led face-to-face interview measure of CA with an independent respondent (mother or significant care giver) that we argue is more sensitive in detecting adversity and less prone to retrospective recall biases than self-report measures. A recent meta-analysis has suggested that such comprehensive interview-based measures of the early environment are more sensitive for detecting putative 5-HTTLPR by environment interactions than self-reported life-stress measures (Karg et al., 2011). Third we included measures of RNLE (that included an assessment of personal negative impact) and of current mood at the time of scanning; and finally we carefully assessed previous lifetime psychiatric history. We excluded s/l participants, since there is some variation in group classification in 5-HTTLPR studies, e.g. the s/l group being classified differently in different studies [for example (Hariri et al., 2002; Lee and Ham, 2008)]. By utilizing such detailed participant classification this study provides greater theoretical precision to the moderating effects of 5-HTTLPR gene variation, CA, RNLE, PH and current mood symptoms on individual differences in amygdala reactivity.

In addition, we wished to functionally investigate neural regions outside our amygdala region of interest. Putative neural regions proposed to also be sensitive to early adverse experiences include the prefrontal cortex, cerebellum and visual cortices (McCrory et al., 2010; Teicher et al., 2003). The negative impact of CA occurring in infancy and childhood may be demonstrable in early and rapidly developing sensory brain areas, whereas later effects of CA may occur in cortical association cortices (Lenroot et al., 2009). For example, grey matter volume is reduced in women with a history of abuse before 12 years of age compared to controls in the left fusiform, left middle occipital, and right lingual gyri (Tomoda et al., 2009). We conjectured that by late adolescence sensory brain regions receiving feedback projections from the amygdala (Amaral et al., 2003) may be functionally altered by exposure to adversities in infancy and childhood. We therefore conducted a further whole-brain investigation with the aim of identifying putative associations with CA potentially moderated by 5-HTTLPR as few studies of CA have included putative genetic moderation of CA on brain function.

## Methods and materials

### Participant recruitment

Participants (*N* = 67; Mean [*SD*] age = 18.6 [0.67]; 31 females) were a subset from the ROOTS study, a longitudinal study of adolescent development (Goodyer et al., 2010). Participants were recruited through secondary schools. The aim of the ROOTS project is to determine the relative contributions of specific genetic, physiological, psychological and social variables to the overall risk for psychopathology emerging during adolescence. We generated a list of all potential ROOTS participants who were eligible based on 5-HTTLPR genotyping and classification of CA using the Cambridge Early Experience Interview (CAMEEI) (see below) (Dunn et al., 2011).

The study coordinator (NDW) attempted to contact 152 eligible participants, being either s/s or l/l homozygotes of the 5-HTTLPR allele, and either having (CA+) or not having (CA−) a history of childhood adversity (see below). Of these, 117 were contactable and 96 agreed to be asked the eligibility questions after being informed about the aims of the study. After a description of the study, participants underwent a brief phone screen to ascertain eligibility as defined by the inclusion/exclusion criteria. Eighty-seven participants proved eligible following this phone screen. If participants were eligible invitation letters and information sheets were sent to interested participants. Following this, an initial assessment and training session were arranged during which participants gave written informed consent. Of those eligible, 80 were recruited to the study. Of this number 71 participants completed the scan session and of this number 67 participants had usable data, described in the present study.

Participants recruited to the neuroimaging sub-study showed no significant selection bias compared to the total ROOTS sample in terms of gender ratio, number of stressful life events experienced aged 16–17 or socioeconomic status as assessed using the ACORN measure (http://www.caci.co.uk). However, participants in the neuroimaging sub-study had lower levels of self-reported depressive symptoms as measured by the Mood and Feelings Questionnaire (MFQ) (Angold et al., 1995) relative to the overall ROOTS sample. This is likely due to differences in inclusion/exclusion criteria between the two samples (i.e. presence of current psychopathology was an exclusion criteria) and age (see (St Clair et al., 2012) for more on this). This comparison information is displayed in Table 1.

The study was carried out in accordance with the Declaration of Helsinki and Good Clinical Practice guidelines. The Cambridgeshire Research Ethics Committee approved the study. All participants provided written informed consent. Inclusion criteria were as follows: participation in the ROOTS study; normal or corrected-to-normal vision; Northern European descent; English speaking. Exclusion criteria were: any history of neurological trauma resulting in loss of consciousness; current psychotropic medication use; current neurological disorder; current DSM-IV Axis 1 disorder; presence of metal in body; specific learning disability, and IQ < 85 (estimated using the Wechsler Abbreviated Scale of Intelligence (Wechsler, 1999)). A number of participants had received a past psychiatric diagnosis through prior ROOTS K-SADS-PL (Kiddie Schedule for Affective Disorders and Schizophrenia for School-Age Children—Present and Lifetime version (Kaufman et al., 1997)) assessments. Comparison of sample participants with the larger ROOTS cohort is shown in Table 1. We report prior participant DSM-IV diagnoses (PH) in Inline Supplementary Table S1.

Inline Supplementary Table S1

**Table S1:** Participant psychiatric history (PH) as diagnosed using K-SADS.

Group	Gender	Previous disorder
l/l CA +	F	Previous NSSI, affective disorder (MDD), anxiety disorder (Panic disorder)
l/l CA +	F	Previous anxiety disorder (Specific phobia)
l/l CA +	M	Previous anxiety disorder (specific phobia)
l/l CA +	M	Previous NSSI, affective disorder (MDD) anxiety disorder (Anxiety NOS)
l/l CA +	M	Previous NSSI
l/l CA +	M	Previous behavioural disorder (HCI CD, ODD & ADHD)
l/l CA +	F	Previous anxiety disorder (Past panic disorder)
l/l CA-	F	Previous affective disorder (MDD), anxiety disorder (Specific spider phobia)
l/l CA-	M	Previous affective disorder (MDD) Previous anxiety disorder (Panic attack)
l/l CA-	M	Previous NSSI, previous MDD
l/l CA-	M	Previous anxiety disorder (OCD & panic attacks)
s/s CA +	F	Previous NSSI, affective disorder (MDD), anxiety disorder (panic disorder), alcohol abuse
s/s CA +	F	Previous NSSI, affective disorder (MDD), anxiety disorder (panic disorder)
s/s CA +	M	Previous behavioural disorder (CD), affective disorder (MDD), substance abuse
s/s CA +	F	Previous NSSI
s/s CA +	M	Previous NSSI, affective disorder (MDD)
s/s CA +	F	Previous eating disorder
s/s CA +	F	Previous affective disorder (MDD)
s/s CA +	F	Previous affective disorder (MDD)
s/s CA-	M	Previous behavioural disorder (ADHD)
s/s CA-	M	Previous alcohol abuse

**Definitions:** NSSI (non-suicidal self injury), MDD (Major depressive disorder), NOS (not otherwise-specified), HCI (high clinical index), CD (conduct disorder), ODD (oppositional defiant disorder), OCD (obsessive compulsive disorder), ADHD (attention deficit hyperactivity disorder). NSSI is reported although it is not a formal DSM-IV diagnosis.

Inline Supplementary Table S1

### Assessment of childhood adversity (0–11 years)—The Cambridge Early Experiences Interview (CAMEEI)

This semi-structured interview is conducted with the child's primary caregiver and records family-focussed adverse life experiences, child's age at occurrence, duration, and an interviewer assessment of their practical impact on the daily life of the family (see (Dunn et al., 2011) for more information). The current investigation used contextual data from the first eleven years of life to classify adolescents into those exposed (CA+) and not exposed (CA−) to early family adversities. Exposure to an adverse family environment was defined as exposure to abuse (emotional, physical or sexual) and/or significant family discord; occasional physical violence, lack of affectionate warmth, or severe lack of communication between family members.

### Assessment of recent negative life events (RNLE) aged 13–14 and 16–17

At ages 14 and 17, participants in the ROOTS cohort had completed a self-report measure of negative life events and difficulties (modified from (Goodyer et al., 2000)), occurring to them, their family or closest friends over the preceding 12 months. Participants were asked to date these experiences and rate their impact on themselves on a scale from 1 = very pleasant/happy to 5 = very unpleasant/sad/painful. If participants rated either 4 or 5 they were asked to indicate if they felt upset for longer than 2 weeks. From these ratings, two separate summed totals for positive and negative recent life events rated as occurring for longer than 2 weeks were derived. The negative event ratings were used here. We have shown acceptable test-retest reliability for the reporting of recent negative events (κ = 0.75), significant agreement (κ = 0.8) between maternal and adolescent perceptions of occurrence (with the exception of sexual behaviours, which are usually not reported by parents), and high agreement (κ = 0.9) between adolescent self-report and (adult) panel ratings of the degree of negative impact of an undesirable event over the next few weeks.

### Assessment of current psychopathology and mood

Prior to scanning participants were screened to assess current psychopathology using the K-SADS-PL. They also completed two well validated and frequently used self reports of current mood (the Mood and Feelings Questionnaire (MFQ (Angold et al., 1995)) for depression and the Spielberger State Anxiety Inventory (SAI (Spielberger, 1983)).

### Genotyping

DNA was harvested from separate saliva samples (Qiagen, Crawley, UK) and genotyped for 5-HTTLPR. The 5-HTTLPR region was amplified using the primers 5-ATGCCAGCACCTAACCCCTAATGT-3 and 5-GGACCGCAAGGTGGGCGGGA-3, which generates a 419 bp and 375 bp product for the “l” and “s” alleles respectively. The PCR reaction mixture consisted of: 100 ng genomic DNA, 10 mM Tris–HCl (pH 9.0), 1.5 mM MgCl_2_, 50 mM KCl, 0.1% Triton®X-100, 1.25 U *Taq* DNA polymerase, 200 μM dNTPs, 500 nM each of forward and reverse primer and 100 μM 7-Deaza-dGTP in a final reaction volume of 15 μL. The reaction conditions were 98 °C for 7 min, followed by 40 cycles of 96 °C for 30 s, 61 °C for 30 s and 72 °C for 1 min with a final extension stage of 72 °C for 10 min. PCR products were electrophoresed on a 3700 DNA analyser (Applied Biosystems) with semi-automated sizing and genotyping performed using GENESCAN v3.7 and GENOTYPER v3.7 software (Applied Biosystems).

Triallelic genotyping was performed using Taqman methodology on a 7900 Sequence Detection System (Applied Biosystems). A 181 bp fragment was amplified using the primers 5-GCAACCTCCCAGCAACTCCCTGTA-3 and 5-GAGGTGCAGGGGGATGCTGGAA-3. Each reaction contained two fluorogenic probes that are specific for the L_A_ allele (5-6FAM-CCCCCCTGCACCCCCAGCATCCC-3) and the L_G_ allele (5′-VIC- CCCCTGCACCCCCGGCATCCCC-3′). PCR amplification of the DNA was completed using 50 ng DNA, 1× Taqman Universal Mastermix (Applied Biosystems), 500 nM each of forward and reverse primer, 80 nM FAM probe (L_A_ allele) and 100 nM VIC probe (L_G_ allele) in a final reaction volume of 5 μL. PCR amplification conditions were 96 °C for 10 min followed by 40 cycles of 96 °C for 15 s and 69 °C for 1 min. Following PCR amplification, an end-point reading of the fluorescence from each probe was measured, with the relative fluorescence of each probe used to genotype individuals. Genotyping was completed using the Sequence Detection System Software Version 2.1 (Applied Biosystems).

### Image acquisition and preprocessing

Functional MRI scanning was performed on a 3-Tesla unit (Siemens Tim Trio with a head coil gradient set; Siemens, Surrey, England) at the MRC Cognition and Brain Sciences Unit. Whole-brain data were acquired with echo-planar T2-weighted imaging (EPI) sensitive to the blood oxygenation level‐dependent signal contrast (32 axial slices, 3 mm thickness; repetition time, 2000 ms; echo time, 30 ms; voxel size, 3 × 3 × 3 mm). Data were analyzed using statistical parametric mapping software (SPM8). The EPIs were sinc interpolated in time to correct for slice time differences and realigned to the first scan by rigid body transformations to correct for head movements. The mean EPI was computed for each subject and inspected to ensure that none showed excessive signal dropout in the medial temporal cortex and OFC. The EPIs were coregistered and normalized to the T1 standard template in the MNI space (Montreal Neurological Institute) using linear and nonlinear transformations and were smoothed with a Gaussian kernel of full width at half maximum of 8 mm.

### fMRI task

**Fig. 1:**
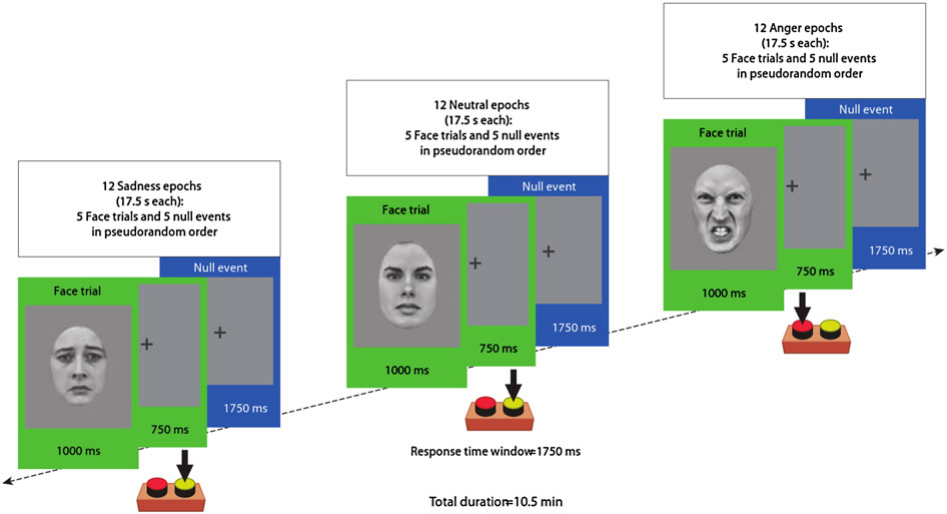
Functional magnetic resonance imaging (fMRI) paradigm and examples of stimuli used (sex discrimination). All participants were shown alternating 17.5‐s epochs containing photographs of angry, sad, or neutral facial expressions (12 epochs of each). Each epoch comprised 5 face trials (green frames) interspersed with 5 null events (fixation cross) (blue frames). A full description of the paradigm is given in the “fMRI” Task subsection of the “Methods and materials” section.

We used an fMRI paradigm known to reliably elicit amygdala reactivity and identical to that reported previously (Passamonti et al., 2010) (see Fig. 1). Participants categorized the sex of grey-scale photographs of angry, sad, and neutral faces (half female) posed by 30 different identities. The faces were selected from 2 stimulus sets (Lundqvist et al., 1998; Tottenham et al., 2009) on the basis of emotional ratings from an independent sample (Ewbank et al., 2009). Emotion ratings were also obtained from participants in the present study after the fMRI session. Stimuli were presented in 17.5-s epochs each containing 5 faces from the same category (angry, sad, or neutral) intermixed with 5 baseline events (fixation cross). Each face trial comprised a 1000-ms presentation of a face followed by a fixation cross (750 ms). Baseline events constituted a 1750-ms presentation of the same fixation cross. The stimuli within each epoch were pseudorandomized with respect to trial type (face or fixation cross) and the face's sex and identity; no more than 3 consecutive trials were of the same trial type. The pseudorandomization enhanced design efficiency while preserving the unpredictability of stimulus onsets in naïve participants. Twelve epochs of each category were presented (60 angry, 60 sad, and 60 neutral faces; total duration, 10 min 30 s).

### Behavioural analyses

Reaction times (RTs) and accuracy were recorded throughout the gender discrimination task. Accuracy and correct RT on the gender discrimination task were submitted to a 2 (Genotype) × 2 (Adversity) × 3 (Valence) mixed-model ANOVA with SAI, RNLE14, RNLE17 and PH included as covariates.

In addition following the scanning session participants were asked to view the face stimuli again and in two runs to classify the faces in terms of either anger or sadness intensity. Emotional ratings and RTs of facial expression ratings obtained after scanning were submitted to a 2 (Genotype) × 2 (Adversity) × 3 (Valence) × 2 (Discrimination Context) mixed-model ANOVA with SAI, RNLE14, RNLE17 and PH included as covariates.

### Neuroimaging data analytic strategy

The proposition under investigation is that adolescents currently free of psychiatric morbidity, but at elevated genetic and/or environmental risk for affective disorders, will show increased amygdala activity compared to those with either one or neither risk factors. We predicted that amygdala reactivity would be greatest as a function of additive genetic and environmental effects as captured by a statistical interaction between being an s/s carrier and exposure to CA. We further hypothesized that this interaction would not be accounted for by PH, RNLE or current mood (depressed or anxious) at the time of scanning.

**Fig. 2:**
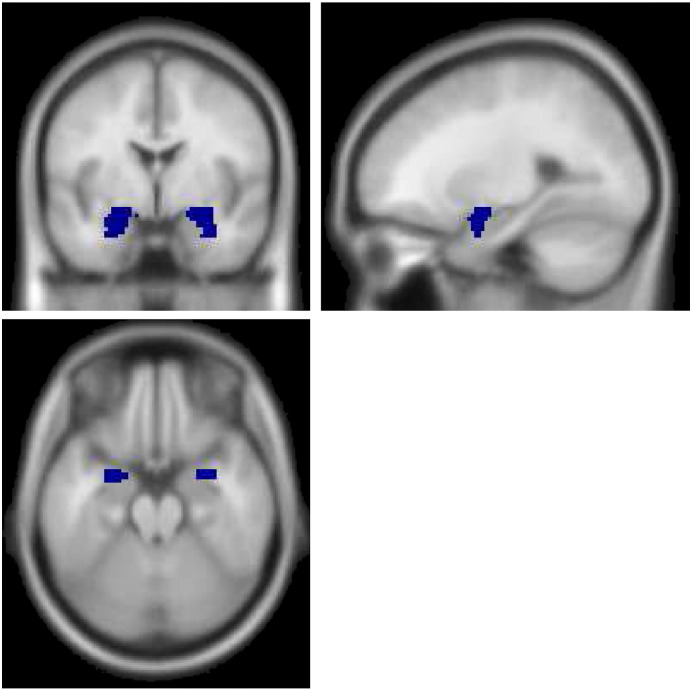
Amygdala ROI derived from the automatic anatomical labeling template.

In order to test our predictions, we performed a similar region of interest analysis to that published previously (von dem Hagen et al., 2011) using an approximate (but not identical) paradigm. First-level t-contrasts of angry, sad and neutral faces (vs. baseline fixation) were extracted in the left and right amygdala from each individual subject using the MARSBAR region of interest software (Brett et al., 2002). Amygdala regions were identified using the Anatomic Automatic Labeling (AAL) atlas (Tzourio-Mazoyer et al., 2002) (see Fig. 2). Extracted values represent mean contrast values over all voxels in either the left of right amygdala. Two subsequent 2 (genotype: s/s, l/l) × 2 (childhood adversity: CA+, CA−) × 3 (valence: anger, sad, neutral) mixed model MANOVAs (including both left and right amygdalae activations) were then implemented in PASW V18. The first MANOVA included no covariates, the second MANCOVA covaried mood (the SAI or MFQ), RNLE14, RNLE17, and PH to disaggregate the effects of CA, genotype, and their interaction on amygdala reactivity from these potential confounds. We also ran additional analyses classifying participants according to the triallelic 5HTTLPR classification (see supplemental results).

Finally, we ran a genotype × CA × valence whole-brain analysis to identify additional brain regions demonstrating genetic and/or environmental moderation. This was run in the same manner as the region of interest analysis above, except first-level contrasts were run in a second level SPM full-factorial group analysis. We discuss only regions surviving strict correction for multiple comparisons at the level of p < 0.05 Family-Wise Error (FWE) corrected. However, to aid future replication studies we report significant regions at p < 0.001 and a 30 voxel threshold.

## Results

### Description of participants

We had four groups of participants categorized according to genotype and presence/absence of CA. There were no significant main or interactive effects of genotype/CA with respect to age, IQ, gender ratio, SAI, or number of upsetting or neutral/pleasant life events aged 16–17. L/l genotype group was associated with significantly higher negative life events aged 13–14 relative to the s/s group (F_1_,_62_ = 5.162, p = .027, η_p_^2^ = 0.08). Presence of adversity (CA+) was associated with significantly more lifetime diagnoses of psychiatric disorder (F_1_,_63_ = 6.84, p = .011, η_p_^2^ = 0.10), and a trend for higher self reported depressive symptoms (F_1_,_63_ = 3.96, p = .051, η_p_^2^ = 0.02) relative to the CA− groups (see Table 2).

### Participant questionnaire associations

The correlations between the independent variables are given in full in the supplemental results (see Inline Supplementary Table S2). In brief there were the expected significant associations between CA and depressive symptoms (MFQ) (n = 67, r = 0.24, p = 0.050) and PH (n = 67, r = 0.31, p = 0.011); RNLE17 and SAI (n = 58, r = 0.42, p = 0.024); SAI and MFQ (n = 58, r = 0.30, p = 0.024), and between MFQ and PH (n = 67, r = 0.269, p = 0.028). RNLE14 was associated with Genotype (n = 66, r = − 0.259, p = 0.036) and PH (n = 66, r = 0.30, p = 0.016).

Inline Supplementary Table S2

**Table S2:** Correlation matrix of participant variables and amygdala reactivity.

Variable	Genotype	CA	RNLE14	RNLE17	SAI	MFQ	PH	LA Anger	LA Sad	LA Neutral	RA Anger	RA Sad	RA Neutral
**Genotype Pearson correlation**	1	.044	− .259	− .021	.061	.033	.010	.288*	.152	.316**	.163	− .002	.266*
**Sig. (2-tailed)**		.725	.036	.872	.628	.789	.938	.018	.219	.009	.188	.988	.030
**N**	67	67	66	60	65	67	67	67	67	67	67	67	67
**CA Pearson correlation**	.044	1	.144	.210	.031	.241*	.310*	− .014	− .040	.070	.132	.027	.148
**Sig. (2-tailed)**	.725		.249	.107	.806	.050	.011	.911	.748	.572	.285	.829	.233
**N**	67	67	66	60	65	67	67	67	67	67	67	67	67
**RNLE14 Pearson correlation**	− .259*	.144	1	− .102	.039	− .019	.296*	− .097	− .087	− .164	− .137	− .082	− .093
**Sig. (2-tailed)**	.036	.249		.438	.762	.878	.016	.438	.489	.189	.272	.515	.457
**N**	66	66	66	60	64	66	66	66	66	66	66	66	66
**RNLE17 Pearson correlation**	− .021	.210	− .102	1	.297*	.175	− .160	.287*	.323	.333**	.219	.193	.263*
**Sig. (2-tailed)**	.872	.107	.438		.024	.182	.223	.026	.012	.009	.093	.140	.042
**N**	60	60	60	60	58	60	60	60	60	60	60	60	60
**SAI Pearson correlation**	.061	.031	.039	.297*	1	.420**	.078	.248*	.339**	.247*	.215	.260*	.178
**Sig. (2-tailed)**	.628	.806	.762	.024		.000	.536	.047	.006	.047	.085	.037	.155
**N**	65	65	64	58	65	65	65	65	65	65	65	65	65
**MFQ Pearson correlation**	.033	.241*	− .019	175	.420**	1	.269*	.100	.197	.252*	.103	.144	.269*
**Sig. (2-tailed)**	.789	.050	.878	.182	.000		.028	.419	.110	.040	.406	.246	.028
**N**	67	67	66	60	65	67	67	67	67	67	67	67	67
**PH Pearson correlation**	.010	.310*	.296*	− .160	.078	.269*	1	− .200	− .216	− .089	− .143	− .126	.001
**Sig. (2-tailed)**	.938	.011	.016	.223	.536	.028		.105	.080	.474	.250	.311	.995
**N**	67	67	66	60	65	67	67	67	67	67	67	67	67
**LA Anger Pearson correlation**	.288*	− .014	− .097	.287*	.248*	.100	− .200	1	.718**	.683**	.844**	.584**	.625**
**Sig. (2-tailed)**	.018	.911	.438	.026	.047	.419	.105		.000	.000	.000	.000	.000
**N**	67	67	66	60	65	67	67	67	67	67	67	67	67
**LA Sad Pearson correlation**	.152	− .040	− .087	.323*	.339**	.197	− .216	.718**	1	.674**	.616**	.831**	.596**
**Sig. (2-tailed)**	.219	.748	.489	.012	.006	.110	.080	.000		.000	.000	.000	.000
**N**	67	67	66	60	65	67	67	67	67	67	67	67	67
**LA Neutral Pearson correlation**	.316**	.070	− .164	.333*	.247*	.252*	− .089	.683**	.674**	1	.543**	.558**	.820**
**Sig. (2-tailed)**	.009	.572	.189	.009	.047	.040	.474	.000	.000		.000	.000	.000
**N**	67	67	66	60	65	67	67	67	67	67	67	67	67
**RA Anger Pearson correlation**	.163	.132	− .137	.219	.215	.103	− .143	.844**	.616**	.543**	1	.713**	.684**
**Sig. (2-tailed)**	.188	.285	.272	.093	.085	.406	.250	.000	.000	.000		.000	.000
**N**	67	67	66	60	65	67	67	67	67	67	67	67	67
**RA Sad Pearson correlation**	− .002	.027	− .082	.193	.260*	.144	− .126	.584**	.831**	.558**	.713**	1	.675**
**Sig. (2-tailed)**	.988	.829	.515	.140	.085	.246	.311	.000	.000	.000	.000		.000
**N**	67	67	66	60	65	67	67	67	67	67	67	67	67
**RA Neutral Pearson correlation**	.266*	.148	− .093	.263*	.260*	.269*	.001	.625**	.596**	.820**	.684**	.675**	1
**Sig. (2-tailed)**	.030	.233	.457	.042	.037	.028	.995	.000	.000	.000	.000	.000	
**N**	67	67	66	60	65	67	67	67	67	67	67	67	67

Definitions: CA (childhood adversity), RNLE (recent negative life events) aged 14 or 17, PH (psychiatric history, SAI (Spielberger Anxiety Inventory), MFQ (Mood and Feelings Questionnaire), LA (left amygdala), RA (right amygdala).

Inline Supplementary Table S2

### Behavioural results

#### On-line gender discrimination results

There were no significant main effects of 5-HTTLPR genotype or CA or 5-HTTLPR × CA interactions upon on-line gender discrimination.

#### Off-line emotion intensity ratings

There were no significant main effects of 5-HTTLPR genotype or CA or 5-HTTLPR × CA interactions upon off-line facial emotion intensity discrimination.

### Amygdala reactivity

#### Effect of 5-HTTLPR genotype and CA upon amygdala reactivity

Our first MANOVA without covariates included a significant multivariate effect of genotype upon amygdala reactivity (Wilks' Lambda = 0.89, F_2_,_62_ = 3.78, p = 0.028, η_p_^2^ = 0.11). No other multivariate terms were significant (see Inline Supplementary Table S3 for the full output). In particular, there were no significant main or interactive effects involving CA (Fs < 1.38, Ps > 0.26).

Inline Supplementary Table S3

**Table S3:** MANOVA 1 (No covariates).

**ANALYSIS 1**					
**Valence x Genotype x CA MANOVA** **(No covariates)**					
**Multivariate tests**					
**Effect**					
**Between Subjects**		**df**	**F**	**p**	**η**_**p**_^**2**^
Genotype⁎		2,62	3.79	0.03	0.11
CA			1.38	0.26	0.04
Genotype x CA			0.20	0.82	0.01
**Within Subjects**		**df**	**F**	**p**	**η**_**p**_^**2**^
Valence		2,64	2.19	0.08	0.13
Valence x Genotype			1.88	0.13	0.11
Valence x CA			0.96	0.44	0.06
Valence x Genotype x CA			0.86	0.49	0.05
**Univariate tests**					
**Within Subjects**	**Hemisphere**	**df**	**F**	**p**	**η**_**p**_^**2**^
Valence⁎	L	2,126	3.78	0.03	0.06
	R		3.08	0.05	0.05
Valence x Genotype⁎	L		1.62	0.20	0.03
	R		3.86	0.02	0.06
Valence x CA	L		0.67	0.51	0.01
	R		0.73	0.49	0.01
Valence x Genotype x CA	L		0.25	0.78	0.00
	R		0.31	0.74	0.01
**Between Subjects**	**Hemisphere**	**df**	**F**	**p**	**η**_**p**_^**2**^
Genotype⁎	L	1,63	5.53	0.02	0.08
	R		1.47	0.23	0.02
CA	L		0.00	0.97	0.00
	R		0.73	0.40	0.01
Genotype x CA	L		0.08	0.78	0.00
	R		0.00	0.95	0.00

Abbreviations: CA (Childhood adversity), RNLE (Recent Negative Life Events), SAI (Spielberger Anxiety Inventory).

⁎ Significant result at p < 0.05.

Inline Supplementary Table S3

We examined the univariate output for the significant main effect of genotype. Data for the left amygdala revealed a main effect of genotype (F_1_,_63_ = 5.53, p = 0.022, η_p_^2^ = 0.08), whereby amygdala reactivity was higher for s/s compared to l/l individuals. Results for the right amygdala were non-significant (F_1_,_63_ = 1.47, p = 0.23, η_p_^2^ = 0.02).

#### Effect of 5-HTTLPR genotype and CA upon amygdala reactivity with covariates for RNLE, current mood and PH

The MANCOVA including RNLE14, RNLE17, SAI, and PH as covariates, confirmed the multivariate effect of genotype (Wilks' Lambda = 0.88, F_2_,_49_ = 3.32, p = 0.044, η_p_^2^ = 0.12). Due to missing data, this analysis was performed with 9 fewer participants, 7 participants had missing RNLE17 data, 2 participants had missing SAI data). There were no other significant multivariate main effects or interactions involving genotype, CA, or any of the covariates (see Inline Supplementary Table S4 for the full output). In particular, there were again no main or interactive effects of CA (Fs < 1.34, ps > 0.27). There was a trend for a multivariate effect of RNLE17, F_2_,_49_ = 2.75, p = 0.074, η_p_^2^ = 0.10, with higher RNLE17 ratings associated with greater amygdala reactivity.

Inline Supplementary Table S4

**Table S4:** MANOVA 2 (with RNLE14, RNLE17, SAI and PH covariates).

**ANALYSIS 2**					
**Valence x Genotype x CA MANOVA** **(with RNLE14, RNLE17, SAI, PH covariates)**					
					
**Multivariate tests**					
**Effect**					
**Between Subjects**		**df**	**F**	**p**	**η**_**p**_^**2**^
Genotype⁎		2,49	3.32	0.04	0.12
CA			1.14	0.32	0.04
Genotype x CA			0.26	0.77	0.01
RNLE14			0.32	0.73	0.01
RNLE17			2.73	0.08	0.10
SAI			0.92	0.40	0.04
PH			1.30	0.29	0.05
**Within Subjects**		**df**	**F**	**p**	**η**_**p**_^**2**^
Valence		4,48	0.70	0.60	0.06
Valence x Genotype			1.51	0.22	0.11
Valence x CA			1.60	0.19	0.12
Valence x Genotype x CA			0.60	0.66	0.05
Valence x RNLE14			1.33	0.27	0.10
Valence x RNLE17			0.46	0.77	0.37
Valence x SAI			0.70	0.60	0.06
Valence x PH			0.73	0.58	0.06
**Univariate tests**					
**Within Subjects**	**Hemisphere**	**df**	**F**	**p**	**η**_**p**_^**2**^
Valence	L	2,100	1.27	0.29	0.03
	R		0.74	0.48	0.02
Valence x Genotype	L		0.53	0.59	0.01
	R		2.60	0.08	0.05
Valence x CA	L		0.26	0.80	0.01
	R		0.61	0.53	0.01
Valence x Genotype x CA	L		0.07	0.93	0.00
	R		0.14	0.87	0.00
Valence x RNLE14	L		0.29	0.75	0.01
	R		0.45	0.64	0.01
Valence x RNLE17	L		0.20	0.82	0.00
	R		0.75	0.48	0.02
Valence x SAI	L		1.02	0.37	0.02
	R		0.96	0.39	0.02
Valence x PH	L		0.70	0.50	0.01
	R		1.15	0.32	0.02
**Between Subjects**	**Hemisphere**	**df**	**F**	**p**	**η**_**p**_^**2**^
Genotype⁎	L	1,50	3.97	0.05	0.07
	R		0.54	0.47	0.01
CA	L		0.06	0.84	0.00
	R		1.01	0.31	0.02
Genotype x CA	L		0.04	0.84	0.00
	R		0.31	0.58	0.01
RNLE14	L		0.00	1.00	0.00
	R		0.20	0.66	0.00
RNLE17⁎	L		4.08	0.05	0.08
	R		0.99	0.30	0.02
SAI	L		1.87	0.18	0.04
	R		1.45	0.23	0.03
PH	L		2.33	0.13	0.05
	R		1.03	0.32	0.02

Abbreviations: CA (Childhood adversity), RNLE (Recent Negative Life Events), SAI (Spielberger Anxiety Inventory), PH (Psychiatric history).

⁎ Significant result at p < 0.05.

Inline Supplementary Table S4

**Figure S1:**
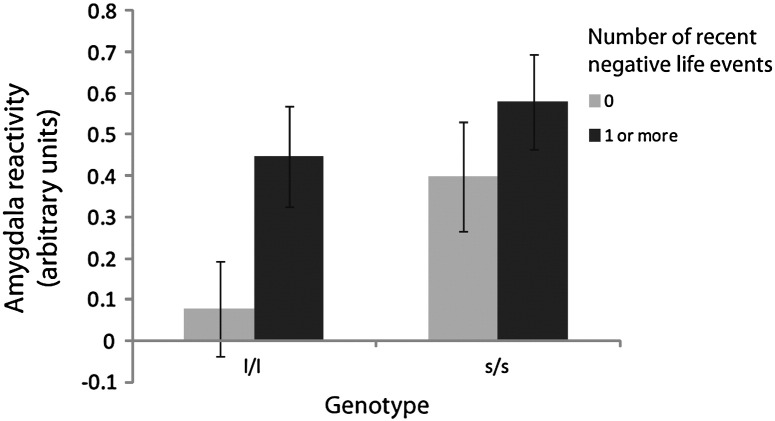
Additive effects of 5-HTTLPR genotype and recent negative life events (RNLE) upon amygdala reactivity. This figure was generated by performing a median split on RNLE to form two groups that either had or had not experienced RNLE.

**Fig. 3:**
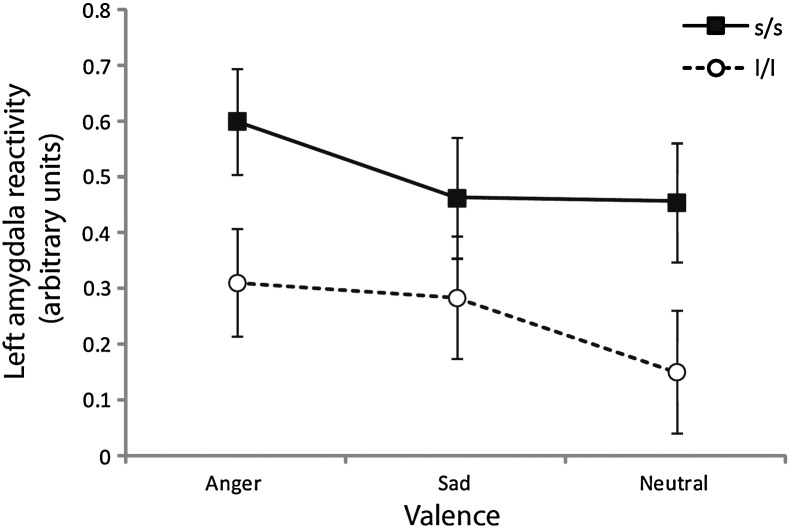
Left and right amygdala reactivity (RNLE14, RNLE17, SAI, and PH covaried).

In the univariate results the main effect of genotype was again significant for the left amygdala (F_1_,_50_ = 3.97, p = 0.05, η_p_^2^ = 0.07) (see Fig. 3), but not the right (F < 1). There was also a main effect of RNLE17 in the left amygdala (F_1_,_50_ = 4.08, p = 0.049, η_p_^2^ = 0.08) but not the right (F_1_,_50_ = 0.99, p = 0.32, η_p_^2^ = 0.02). Results were identical when sum scores for depressed mood on the MFQ were substituted for anxiety symptoms on the SAI. The significant additive effects of genotype and RNLE17 in the left amygdala are presented for illustrative purposes in Fig. S1.

### Whole-brain reactivity

An identical whole-brain analysis was run to the analyses described above. We first ran an analysis with no covariates. This identified a significant main effect of genotype (p < 0.05 FWE) in a cluster in the left cuneus (BA17), whereby s/s individuals showed greater neural reactivity compared to l/l individuals. We also found a main effect of CA in a cluster in the right cuneus (BA19) where CA+ individuals demonstrated reduced neural reactivity compared to CA− individuals. Finally we found a genotype × CA interaction in one cluster located in the left lingual gyrus (BA17). These results are reported in full in Inline Supplementary Table S5. Post-hoc analyses demonstrated that this interaction was due to s/s and l/l CA+ individuals showing no difference in neural reactivity in this region, whereas s/s CA− individuals demonstrated significantly higher neural reactivity compared to l/l individuals.

Inline Supplementary Table S5

**Table S5:** Whole brain effects (no covariates): threshold at p < 0.01, 30 voxel cluster threshold.

Comparison	Region	Cluster size (k_E_)	p (FWE-corr)	F	Z	X	Y	Z
*Main effect of genotype*	*Cuneus (BA17)	992	.008	28.05	4.97	− 6	− 98	0
			.142	20.71	4.28	− 28	− 66	− 16
			.276	18.84	4.07	− 16	− 98	6
	Cerebellum	185	.291	18.68	4.06	32	− 50	− 26
	Inferior occipital gyrus (BA18)	58	.598	16.25	3.77	36	− 76	− 6
	Inferior temporal gyrus (BA37)	37	.724	15.39	3.67	− 36	− 70	− 2
	Cerebellum	71	.763	15.11	3.63	44	− 62	− 28
*Main effect of CA*	*Cuneus (BA19)	128	.012	27.04	4.89	30	− 92	26
	Cuneus (BA19)	271	.054	23.22	4.53	− 22	− 96	28
			.068	22.64	4.47	− 20	− 102	18
			.343	18.19	4.00	− 26	− 88	12
*Genotype x CA interaction*	*Lingual gyrus (BA17)	68	.023	25.35	4.73	− 20	− 84	− 4

* Significant at p < 0.05 Family-wise error corrected.

Inline Supplementary Table S5

These results were then re-run with the inclusion of RNLE14, RNLE17, SAI and PH covariates. These results are reported in full in Table 3.

**Fig. 4:**
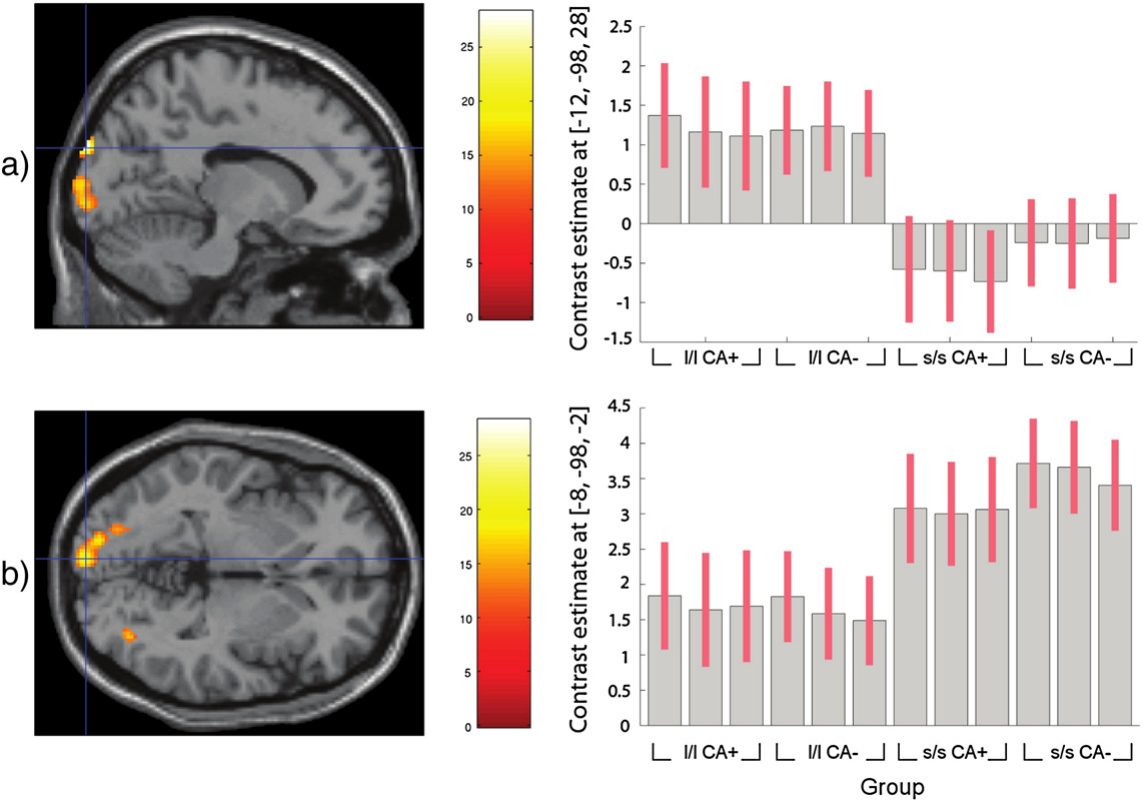
Whole-brain imaging results (RNLE14, RNLE17, SAI, and PH covaried): main effect of genotype. a) Peak cluster at left cuneus (BA19) [Montreal Neurological Institute coordinates − 12,− 98,28] demonstrating reduced activation in s/s compared to l/l group. Activation overlaid on axial section, thresholded at p < 0.001, 30 voxel threshold. b) cluster at left cuneus (BA17) [Montreal Neurological Institute coordinates − 8,− 98,2] demonstrating enhanced activation in s/s compared to l/l group. Activation overlaid on axial section, thresholded at p < 0.001, 30 voxel threshold. In both figures the intensity bar represents F score and graph bars represent the contrast estimates and red lines represent 90% confidence intervals. The three bars for each group represent brain activation to anger, sad and neutral face conditions respectively. CA+ and CA− signify the presence or absence of childhood adversity; l/l and s/s refer to genotype groups.

The effect of genotype in the left cuneus (BA17) reported above, whereby s/s individuals demonstrated higher neural reactivity compared to l/l individuals, was reduced (p = 0.099 FWE corrected) following inclusion of the covariates (see Fig. 4b). However, a more dorsal region of the left cuneus (BA19) demonstrated significantly higher activation in l/l compared to s/s individuals (p = 0.008 FWE corrected) (see Fig. 4a). This change in the pattern of activation is likely due to the effect of genotype on RNLE14 score (see Participant questionnaire associations section above).

**Fig. 5:**
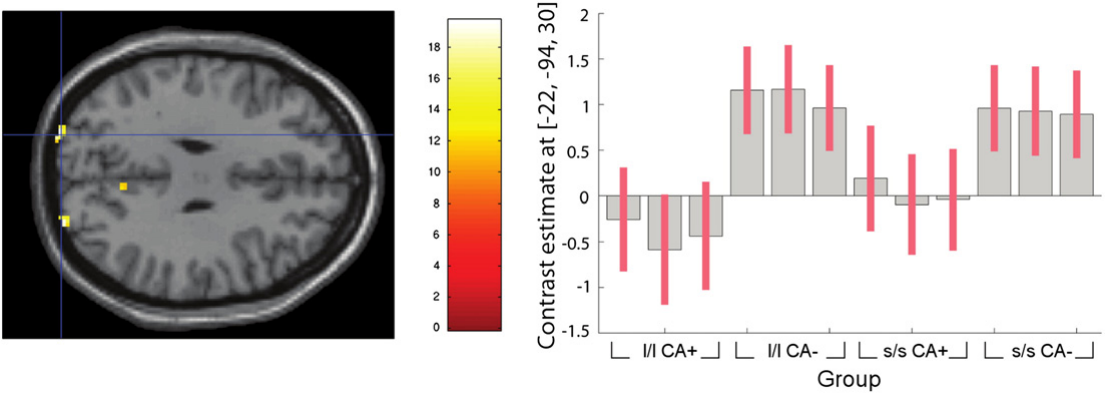
Whole-brain imaging results (RNLE14, RNLE17, SAI, and PH covaried): main effect of CA. Left panel: peak cluster at left cuneus (BA19) [Montreal Neurological Institute coordinates − 22,− 94, 30] overlaid on axial section, thresholded at p < 0.001, 30 voxel threshold. Intensity bar represents F score. Right panel: graphical representation of CA main effect. Bars represent the contrast estimates and red lines represent 90% confidence intervals. The three bars for each group represent brain activation to anger, sad and neutral face conditions respectively. CA+ and CA− signify the presence or absence of childhood adversity; l/l and s/s refer to genotype groups.

However the main effect of CA was no longer significant at p < 0.05 FWE but rather demonstrated a trend towards significance (see Fig. 5 and Table 3).

**Figure S2:**
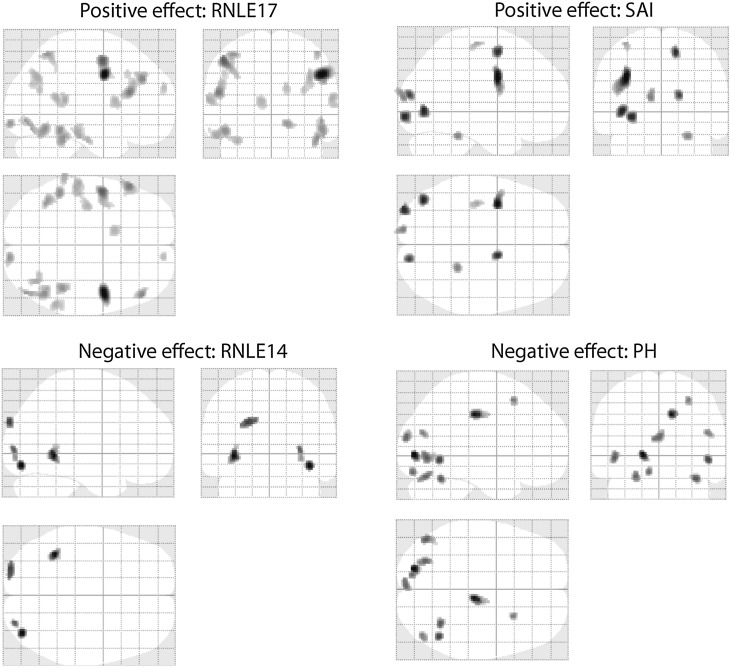
Glass-brain rendering of covariate effects: recent negative life events aged 14 (RNLE14) and aged 17 (RNLE17); Spielberger state anxiety inventory scores (SAI) and psychiatric history (PH). Note RNLE17 and SAI show a significant positive effect on brain reactivity, in contrast RNLE14 and PH shows a significant negative effect on brain reactivity.

This reduction in statistical significance of the main effect of CA may be due to the fewer subjects in this analysis, the addition of covariates, and the strong association, and therefore potential confounding of CA and PH. To aid understanding, we report the whole-brain effects of RNLE14, RNLE17, SAI and PH covariates in Fig. S2 and Inline Supplementary Table S6. These covariate results demonstrate that RNLE17 and SAI had a broad effect of increasing visual cortex reactivity to all faces, whereas RNLE14 and PH had a broad effect of reducing visual cortex reactivity. Thus the inclusion of the RNLE14 and PH covariates reduced the ability to detect significant effects of CA upon neural reactivity.

Inline Supplementary Table S6

**Table S6:** Whole-brain effects of RNLE14, RNLE17, SAI and PH covariates.

Comparison	Region	Cluster size (k_E_)	p (FWE-corr)	T	Z	X	Y	Z
*Positive effect of RNLE14*		75	.087	4.51	4.36	− 30	− 68	52
*Negative effect of RNLE14*		48	.288	4.12	4.01	38	− 82	− 14
		89	.320	4.08	3.97	− 38	− 50	− 4
		69	.621	3.78	3.69	− 24	− 94	28
		40	.736	3.67	3.59	28	− 90	2
			.919	3.43	3.37	30	− 88	− 6
*Positive effect of RNLE17*	* Precentral gyrus (BA6)	226	< .001	6.91	6.45	48	− 2	38
	* Precentral gyrus (BA6)	182	.007	5.19	4.98	− 50	− 4	50
			.687	3.72	3.63	− 38	0	38
	* Inferior frontal gyrus (BA45)	177	.041	4.72	4.56	− 54	24	20
			.570	3.82	3.73	− 52	28	28
	Middle frontal gyrus (BA46)	74	.098	4.47	4.33	50	36	32
	Fusiform gyrus (BA37)	139	.144	4.35	4.22	48	− 60	− 16
			.271	4.14	4.03	48	− 80	− 8
			.376	4.02	3.91	48	− 74	− 16
	Fusiform gyrus (BA37)	90	.152	4.34	4.21	44	− 42	− 24
	Fusiform gyrus (BA37)	74	.154	4.33	4.20	− 48	− 46	− 18
	Inferior parietal lobule (BA40)	83	.195	4.25	4.13	− 40	− 58	56
			.969	3.31	3.25	− 40	− 50	42
	Lingual gyrus (BA18)	58	.201	4.24	4.12	14	− 96	− 10
	Inferior parietal lobule (BA40)	33	.354	4.04	3.93	44	− 58	58
	Inferior temporal gyrus (BA20)	186	.362	4.03	3.93	− 58	− 26	− 22
			.579	3.82	3.73	− 36	− 18	− 28
			.586	3.81	3.72	− 48	− 26	− 18
	Precuneus (BA39)	116	.401	3.99	3.89	36	− 68	34
			.790	3.61	3.53	30	− 66	22
	Superior temp. gyrus (BA22)	68	.454	3.93	3.89	− 64	− 40	12
	Superior temp. gyrus (BA22)	94	.464	3.93	3.83	60	− 50	14
	Caudate	92	.563	3.83	3.74	− 12	10	12
	Superior frontal gyrus (BA10)	35	.667	3.73	3.65	14	58	24
*Negative effect of RNLE17*	Insula (BA13)	61	.608	3.79	3.70	40	12	14
*Positive effect of SAI*	*Precentral gyrus (BA6)	214	.031	4.80	4.63	− 38	− 2	30
	Middle occipital gyrus (BA37)	96	.105	4.45	4.31	− 40	− 76	0
	Middle occipital gyrus (BA18)	85	.120	4.41	4.28	− 32	− 96	− 6
	Middle frontal gyrus (BA6)	74	.130	4.38	4.25	12	− 2	56
	Middle occipital gyrus (BA18)	61	.214	4.22	4.10	16	− 90	14
	Middle occipital gyrus (BA18)	58	.605	3.79	3.70	− 12	− 96	12
	Cerebellum	50	.648	3.75	3.67	24	− 42	− 26
	Cerebellum	30	.865	3.52	3.45	− 38	− 22	64
*Negative effect of SAI*	No significant clusters							
*Positive effect of PH.*	No significant clusters							
*Negative effect of PH*	Lingual gyrus (BA18)	54	.233	4.19	4.08	− 18	− 84	− 2
	Cingulate gyrus (BA24)	103	.289	4.12	4.01	10	− 24	38
			.975	3.29	3.23	14	− 10	38
	Inferior temporal gyrus (BA19)	42	.580	3.82	3.73	48	− 60	− 6
	Cerebellum	43	.674	3.73	3.64	34	− 58	− 24
	Inferior temporal gyrus (BA37)	82	.678	3.72	3.64	− 46	− 72	− 4
	Cuneus (BA18)	66	.727	3.68	3.60	− 2	− 92	16
	Cerebellum	59	.731	3.67	3.59	− 26	− 72	− 22
		31	.753	3.65	3.57	− 12	− 86	− 18
		31	.815	3.58	3.51	28	16	62
	Middle temporal gyrus (BA39)	54	.642	3.76	3.67	46	− 76	20

* Significant at p < 0.05 Family-wise error corrected for multiple comparisons. RNLE = Recent Negative Life Events, SAI = Spielberger State Anxiety Inventory, PH = Psychiatric History.

Inline Supplementary Table S6

**Fig. 6:**
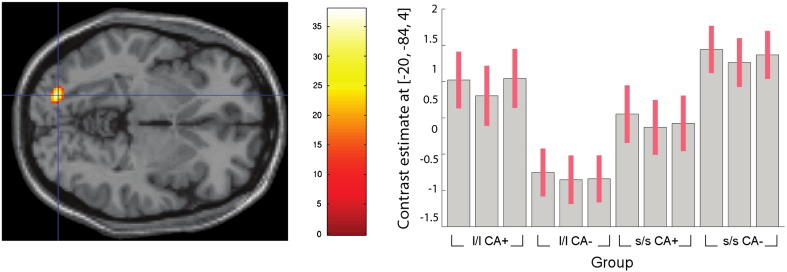
Whole-brain imaging results (RNLE14, RNLE17, SAI, and PH covaried): genotype by CA interaction in left lingual gyrus (BA19) [Montreal Neurological Institute coordinates − 20,− 84,− 4]. Left panel: peak cluster overlaid on axial section, thresholded at p < 0.001, 30 voxel threshold. Intensity bar represents F score. Right panel: Graphical representation of genotype by CA interaction. Bars represent the contrast estimates and red lines represent 90% confidence intervals. The three bars for each group represent brain activation to anger, sad and neutral face conditions respectively. CA+ and CA− signify the presence or absence of childhood adversity; l/l and s/s refer to genotype groups.

In this second whole-brain analysis we again detected a significant genotype by CA interaction in the left lingual gyrus (BA17) (see Fig. 6).

**Fig. 7:**
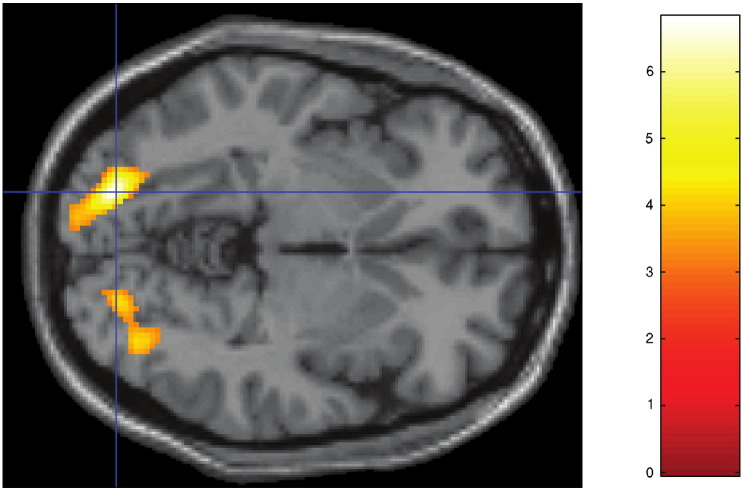
Whole-brain imaging results (RNLE14, RNLE17, SAI, and PH covaried): post-hoc simple main effects test of genotype by CA interaction showing lingual gyrus regions (BA19) [Montreal Neurological Institute coordinates − 20,− 84,− 4] demonstrating increased activation in s/s CA− group compared to l/l CA− group. There was no significant difference between s/s CA+ and l/l CA+ groups. Activation overlaid on axial section, thresholded at p < 0.001, 30 voxel threshold. Intensity bar represents T score.

Post-hoc tests of simple main effects showed that s/s or l/l participants who experienced CA did not differ in neural reactivity in this region, whereas there was a strong genotypic difference in neural activity in those that did not experience CA, with s/s participants being significantly higher in activation than l/l participants (see Fig. 7).

## Discussion

The primary objective of this investigation was to test the hypothesis that the amygdala is a neural locus for a 5-HTTLPR genotype × environment interaction as suggested from population-level association studies. In particular, we sought to elucidate whether exposure to adverse family experiences before the age of 11 years (CA) activates this neural structure known to be associated with emotion processing either as a simple main effect or in interaction with genotype, by disaggregating the effects of variables that have potentially confounded previous studies.

Our results demonstrated clear support for an effect of 5-HTTLPR genetic variation on amygdala reactivity (η_p_^2^ = 0.07, medium effect size). This effect size is slightly larger than current meta-analysis derived estimates of amygdala reactivity that demonstrate a small effect size (Murphy et al., 2012). However, in this most recent meta-analysis it was noted that there is substantial between study heterogeneity that may result from insufficient assessment of environmental factors upon amygdala reactivity. Such insufficient assessment of environmental factors may consequently reduce the sensitivity of detecting genetic effects. In addition our results found no support for either a main effect of CA (η_p_^2^ = 0.001) or a statistical interaction with 5-HTTLPR genotype involving CA (η_p_^2^ = 0.001) upon amygdala reactivity.

This finding of no support for a CA effect is at odds with previous studies showing heightened amygdala reactivity in adolescents and adults exposed to CA (Dannlowski et al., 2012; Grant et al., 2011; Maheu et al., 2010; McCrory et al., 2011; Tottenham et al., 2011). A number of features may account for these differences. For example, the nature of CA assessed across studies may have been qualitatively different (i.e. commission e.g. physical/sexual abuse vs. omission e.g. neglect), or differences in the methods used to determine psychiatric state (i.e. retrospective vs. prospective assessment) that may critically modify participant reports (Moffitt et al., 2010). Therefore amygdala effects attributed to CA in previous studies may have been confounded by participant's genotype, RNLE, anxiety symptoms, or diagnosable psychopathology, given that prior studies did not assess or control all these factors concurrently. Here we excluded participants with any current psychopathology and included these other factors as covariates and indeed found support for an effect of RNLE17 upon left amygdala reactivity with a medium effect size. This supports the notion that proximal rather than distal adverse environmental events such as CA or RNLE14 exert a greater influence on amygdala reactivity. Importantly, these RNLE17 effects were not accounted for by current mood at the time of the investigation. Interestingly, our results are in line with Armbruster et al. (2009) who found increased startle response associated with the s/s genotype and higher recent but not early or cumulative negative life events. This research group also reported an additive association between 5-HTTLPR genotype and RNLE, consistent with the current results (see Fig. S1). The whole-brain analysis did find effects of CA in a more dorsal region of the cuneus (BA19), although this finding was attenuated when covariates were included. Nevertheless, this is consistent with Tomoda and colleagues who reported grey matter reductions in visual cortex in women with a childhood history of abuse (Tomoda et al., 2009).

Turning to the significant neural effects of genotype in the present findings, it is important to note that we found elevated left-sided amygdala and cuneus (BA19) reactivity to *all* face stimuli regardless of the valence of the facial expression, including neutral faces. This result therefore differs from a number of prior amygdala reports and to Thomason et al. (2010) who found greater visual cortex activation to fear compared to neutral faces in s-carriers using a dot-probe task. However, our finding is bolstered by the recent observation of increased visual working memory in s-carriers compared to l/l participants (Anderson et al., 2012). The current findings of increased visual cortex activation may be a neural correlate of such enhanced visual working memory. It is a matter of ongoing debate what affective information these neutral face stimuli convey, as both valence and dominance effects have been demonstrated (Todorov and Engell, 2008; Wright and Liu, 2006). Importantly, a recent meta-analysis of emotion processing studies has reinforced the finding that neutral facial expressions reliably activate the amygdala, suggesting that these stimuli have significant emotional salience (Fusar-Poli et al., 2009). One possibility therefore is that neutral face stimuli provoke activation due to their inherent emotional ambiguity. Additionally from a methodological and psychological perspective, further studies could also usefully determine whether greater amygdala and cortical activation varies as a function of stimulus type, novelty and complexity as we did not include another non-face control stimulus or other sensory methods for evoking emotion (e.g. pain, taste or auditory cues) (Wendt et al., 2011).

In relation to theoretical models of 5-HTTLPR and amygdala reactivity, these results suggest a valence-general, and both environmental and genetically mediated phasic amygdala reactivity, rather than the tonic model of genetically mediated amygdala activation proposed by Canli and others (Canli and Lesch, 2007; Canli et al., 2005, 2006; Heinz et al., 2007) whereby there is a decrease in amygdala activation to neutral faces. One possible reason for the somewhat discrepant findings is that the studies advocating the ‘tonic’ argument have generally used block designs and it may be that the greater length of the fixation block used in these studies increases the degree of unconstrained cognitive activity (Stark and Squire, 2001), and thereby increases the baseline amygdala activity in s/s-allele carriers. In contrast, the briefly presented fixation trials in the present design negated such confounding effects. In addition, previous studies have not taken into account other important confounds of the type that we have sought to control in the present study e.g. the s/s-group in (Canli et al., 2006) were significantly higher in neuroticism as measured at the time of scanning.

As a methodological note, in this study we used the AAL atlas in order to perform our amygdala region-of-interest analysis. The AAL atlas was used as this was the method used in previous recent publications by our group (von dem Hagen et al., 2011) and our associates (Dannlowski et al., 2010, 2012). We chose to use the AAL atlas as we could then perform identical analyses and control for any methodological factors in order to facilitate between-study comparisons. However, we acknowledge recent methodological advances that have led to the development of probabilistic atlases (Devlin and Poldrack, 2007) that more accurately take into account inter-individual variability of brain structure and recommend future studies using region-of-interest analyses take advantage of these methodological advances.

The finding of a 5-HTTLPR by CA interaction in the left lingual gyrus was an unexpected finding that was not predicted on an a priori basis. This finding is parsimoniously best considered as a response to face-processing rather than facial emotion processing as it was independent of valence. The lingual gyrus is commonly activated as part of a face-processing network (Fusar-Poli et al., 2009), however, it is unclear if such an interaction is specific to faces or other complex visual environmental stimuli as no complex control stimuli was included in this experiment. In addition, the 5-HTTLPR × CA interaction finding is a similar finding to the 5-HTTLPR by life-stress interaction observed in left inferior occipital gyrus previously reported by Canli et al. [see Supplementary Table 2 in (Canli et al., 2006)] that was also independent of valence. However, additional replication studies are required to fully establish that this region reliably mediates a 5-HTTLPR by environment interaction effect. Regarding the CA main effect in the dorsal cuneus, it is unclear how CA reduces neural activation in this region. Neither do we know how 5-HTTLPR and CA interact to generate the pattern of activation in the lingual cortex. Our findings may potentially be explained by sustained vigilance towards negative or ambiguous facial expressions in these individuals occurring during the birth to 11 year developmental period when the CA was experienced. In animals, maternal separation is associated with enhanced activation and increased cortisol response in the visual cortex (Rilling et al., 2001). Therefore such sustained activation due to stress (occurring in childhood) that may be adaptive in the short-term may be neurotoxic over the long-term; see (Wilkinson and Goodyer, 2011) for more on such allostatic adaptation. This argument concurs with the covariate effects whereby SAI and RNLE17 covariates increase visual cortex reactivity and the RNLE14 and PH covariate decreased visual cortex reactivity, possibly due to excessive stress associated with PH (see Fig. S2). Alternatively, methylation patterns at the 5-HTTLPR gene occurring as a function of differential environmental experience may also be related to our findings. Increased methylation (of the 5-HTT promoter associated CpG island) has been related to childhood abuse (Beach et al., 2010). Higher levels of methylation among subjects with the l/l genotype was associated with heightened risk of symptoms of unresolved loss or other trauma, whereas higher levels of methylation among subjects with the s/s genotype was associated with diminished risk of these symptoms (van et al., 2010). However, it is unclear why these effects of childhood adversity in our sample are limited to the occipital lobe. It could well be an effect of using only a visual processing task. Other experimental probes may detect functional and structural effects of CA in other brain regions. Delineating causal mechanisms at the neural systems level requires a greater understanding of how these functional effects arise, and how these CA affected regions dynamically interact with other functionally and anatomically connected brain regions. Ideally longitudinal studies using a range of functional tasks and repeat measures designs are needed that investigate gene by environment interactions at the neural systems level.

To date there have been 6 prior studies that have investigated how 5-HTTLPR genotype and environmental factors interact to moderate amygdala and whole-brain reactivity (Alexander et al., 2012; Canli et al., 2006; Drabant et al., 2012; Klucken et al., 2012; Lemogne et al., 2011; Williams et al., 2009). Of these six studies, three have used emotional face-processing paradigms (Alexander et al., 2012; Canli et al., 2006; Williams et al., 2009). Two of these studies (Alexander et al., 2012; Williams et al., 2009) reported that those carrying one or two copies of the s-allele and who reported greater ‘life-stress’ demonstrated greater amygdala reactivity compared to the other groups. One study reported (Canli et al., 2006) reduced amygdala reactivity with increasing life-stress but it was proposed that this was due to an increased baseline of amygdala activity in the risk group. Therefore the results in the current report differ from these prior studies. Another prior study using an emotion regulation paradigm (Lemogne et al., 2011) found a genotype × condition × stress interaction whereby during self-referential processing (compared to an emotion-labelling condition) there was a negative correlation upon amygdala reactivity in the s-carrier group with increasing life stress but a positive correlation in the L group. In a recent study using a fear-conditioning paradigm (Klucken et al., 2012) did not report a 5-HTTLPR × stress interaction in the amygdala but found that the s/s group who reported greater life stress had greater neural reactivity in left occipital and right insula cortex. Finally (Drabant et al., 2012) used an unpredictable shock paradigm and although they did not assess how experienced life stress interacts with neural reactivity, they did report that genotype differentially moderated left medial prefrontal cortex activation and subjective anxiety in response to the stress exposure task. This finding suggests that genotype differentially moderates the neural response to current environmental stressors. This prior literature alongside the present results suggests substantial between-study differences. One tentative finding may be that relative to the l/l group, s/s individuals show greater reactivity to current, or temporally proximate, rather than distal stressors. Whether this neural reactivity occurs in an additive or interactive fashion cannot be fully concluded based on the literature to date. Future studies are critically needed to fully establish the existence of this putative 5-HTTLPR × current stress relationship upon neural reactivity whilst adequately controlling for baseline neural activation levels. This would additionally clarify whether the l/l group show an opposite pattern to the s-carrier group demonstrated through a cross-over interaction in a number of studies (Canli et al., 2006; Drabant et al., 2012; Lemogne et al., 2011).

Other outstanding questions concern the role of distal, proximate or current stress exposure upon neural reactivity. The current results found no effect of distal stress (CA, RNLE14) upon amygdala reactivity but other studies have reported an effect that is differentially moderated by 5-HTTLPR genotype (Williams et al., 2009). Future studies are therefore required to conclusively establish the role of this temporal stress exposure on neural reactivity. It has been shown that those who experience distal adversities are also more likely to experience proximate or current stressors (Liu and Alloy, 2010). This increased susceptibility to stressful events that are at least in part influenced by the individual is known as the ‘stress generation’ model of depression (Hammen, 1991). The present study was underpowered to examine this ‘stress-generation’ model but hypotheses can now be generated that should be tested in future studies. Additionally, the role of regulatory processes such as pre-learnt skills, on-line regulation or learning control processes upon neural reactivity are unclear. Such processes may buffer or prevent stress exposure from becoming uncontrollable and overwhelming. Again, it is currently unclear how distal, proximate or current stress exposures moderate the underlying neural regulatory circuits that may buffer any 5-HTTLPR × recent stress interaction. Finally, it is unknown how 5-HTTLPR × stress exposure moderates task performance. Although 5-HTTLPR × stress exposure has been shown to moderate neural reactivity, it is unclear what the functional consequences of this interaction are. Future studies are therefore required to examine functional effects of 5-HTTLPR × environment interplay on neural reactivity across a range of range of cognitive, emotional and behavioural states.

## Conclusions

In conclusion, our results provide evidence for different forms of 5-HTTLPR by environment interplay. In terms of amygdala reactivity, we demonstrate that proximal, rather than distal adverse experiences exert effects on amygdala reactivity in an additive manner with 5-HTTLPR genotypic variation. On a whole-brain level we found main effects of genotype and CA in ventral and dorsal region of the cuneus respectively and a 5-HTTLPR by CA interaction in the left lingual gyrus. These findings suggest that functional brain topography is differentially affected by genetic and environmental influences.

The following are the supplementary materials related to this article.

Supplementary materials

**Figure S3:**
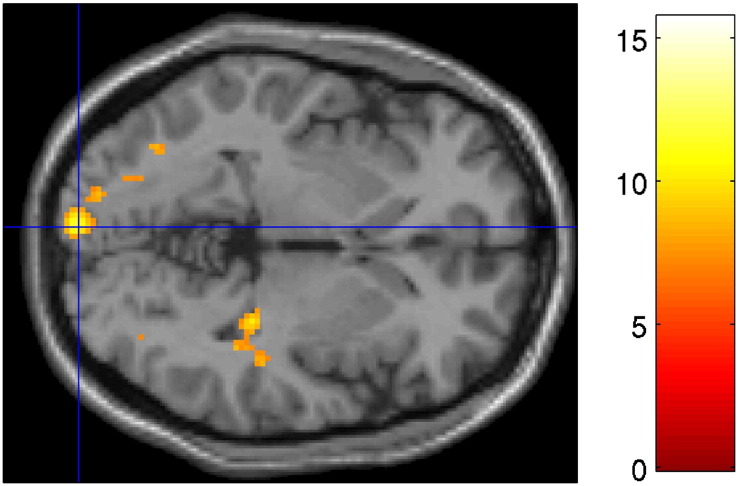
Supplemental confirmatory results of all faces vs. fixation contrast (RNLE14, RNLE17, SAI, and PH covaried): main effect of genotype. Activation in left cuneus (BA17) [Montreal Neurological Institute coordinates − 6,− 98,− 2] overlaid on axial section and thresholded at p < 0.01. Intensity bar represents F score.

**Figure S4:**
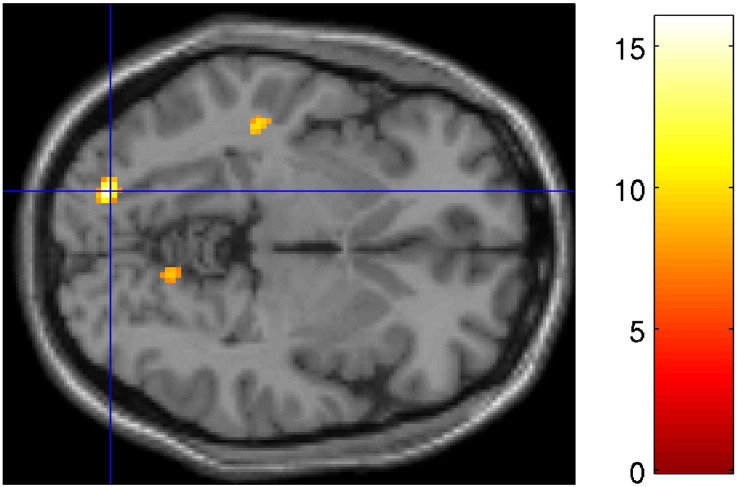
Supplemental confirmatory results of all faces vs. fixation contrast (RNLE14, RNLE17, SAI, and PH covaried): genotype by CA interaction. Activation present in left lingual gyrus (BA19) [Montreal Neurological Institute coordinates − 20,− 84,− 4] overlaid on axial section and thresholded at p < 0.01. Intensity bar represents F score.

## Financial disclosures

Ian Goodyer has received payment from Janssen for lectures.

## Figures and Tables

**Table 1:** Comparison of ROOTS total sample and neuroimaging sample.

Measure	Neuroimaging sample	Total ROOTS sample
N =	67	1143
Depressive symptoms (MFQ)⁎	10.3 (8.3)	14.4 (11.5)
Gender ratio (M/F) %	55/45	46/54
Number of negative life events in previous 12 months	0.86 (1.29)	0.82 (1.22)
Socioeconomic status -		
Wealthy/urban	59%	62%
Comfortable	25%	24%
Moderate means/hard-pressed	16%	14%

⁎ ROOTS sample significantly higher in Mood and Feelings Questionnaire (MFQ) score P < 0.05.

**Table 2:** Participant variables.

	Group			
Variable	l/l CA+	l/l CA−	s/s CA+	s/s CA−
N =	15	19	16	17
Age (Y/M) [Mean (SD)]	18.5 (0.6)	18.6 (0.8)	18.5 (0.5)	18.9 (0.6)
Gender (M/F)	8/7	11/6	7/9	10/7
IQ [Mean(SD)]	108.8 (7.8)	105.7 (10.3)	105.4 (9.7)	106.6 (9.8)
#MFQ [Mean(SD)]	10.9 (9.6)	9.3 (7.0)	13.8 (8.3)	7.5 (7.6)
SAI [Mean(SD)]	33.4 (9.5)	31.4 (9.2)	32.9 (6.7)	33.8 (9.5)
Upsetting life events (2+ weeks) during age 16–17	1.1 (2.1)	0.7 (0.8)	1.2 (1.1)	0.6 (0.6)
Neutral/pleasant life events during age 16–17	1.1 (1.2)	0.7 (0.8)	0.8 (1.3)	1.1 (1.1)
*Upsetting life events (2+ weeks) during age 13–14	0.6 (0.7)	0.3 (0.5)	0.2 (0.4)	0.1 (0.3)
Neutral/pleasant life events during age 13–14	0.7 (0.9)	0.5 (0.7)	0.8 (1.0)	0.8 (0.7)
**Psych. history [Mean (SD)]	0.4 (0.5)	0.2 (0.4)	0.5 (0.5)	0.1 (.03)

# trend for main effect of CA (p = 0.051), *significant main effect of genotype (p = 0.027), **significant main effect of CA (p = 0.011).

**Table 3:** Whole brain results**.**

Comparison	Region	Cluster size (k_E_)	p (FWE-corr)	F	Z	X	Y	Z
*Main effect of genotype*	⁎Cuneus (BA19)	47	0.008	28.20	4.95	− 12	− 98	28
Cuneus (BA17)	477	0.099	21.72	4.35	− 8	− 98	− 2
0.133	20.93	4.27	− 16	− 100	6
0.194	19.87	4.16	− 20	− 90	0
Middle occipital gyrus (BA19)	52	0.367	17.97	3.96	36	− 74	− 4
Lingual gyrus (BA18)	233	0.398	17.70	3.93	− 28	− 66	− 16
		0.454	17.25	3.87	− 24	− 52	− 14
Cerebellum	91	0.424	17.49	3.90	32	− 50	− 26
*Main effect of CA*	Cuneus (BA19)	31	0.209	19.67	4.14	− 22	− 94	30
Cuneus (BA19)	51	0.227	19.42	4.12	28	− 92	28
Precuneus (BA31)	42	0.563	16.45	3.78	8	− 58	26
Middle occipital gyrus (BA19)	70	0.568	16.42	3.78	− 24	− 90	10
0.742	15.19	3.63	− 20	− 100	16
*Genotype* × *CA interaction*	⁎Lingual gyrus (BA19)	115	0.000	37.86	5.70	− 20	− 84	− 4

⁎ Significant at p < 0.05 family-wise error corrected for multiple comparisons.
