# Supplementary material for: 5-HTTLPR–environment interplay and its effects on neural reactivity in adolescents
Source: Neuroimage. 2012 Nov 15;63-248(3):1670–80. doi: 10.1016/j.neuroimage.2012.07.067 (PMC3480648; doi:10.1016/j.neuroimage.2012.07.067)
Supplement: Inline Supplementary Table S1 [file mmc1.docx]

Table S1. Participant psychiatric history (PH) as diagnosed using K-SADS.

| **Group** | **Gender** | **Previous disorder** |
| --- | --- | --- |
| l/l CA+ | F | Previous NSSI, affective disorder (MDD), anxiety disorder (Panic disorder) |
| l/l CA+ | F | Previous anxiety disorder (Specific phobia) |
| l/l CA+ | M | Previous anxiety disorder (specific phobia) |
| l/l CA+ | M | Previous NSSI, affective disorder (MDD) anxiety disorder (Anxiety NOS) |
| l/l CA+ | M | Previous NSSI |
| l/l CA+ | M | Previous behavioural disorder (HCI CD, ODD & ADHD) |
| l/l CA+ | F | Previous anxiety disorder (Past panic disorder) |
| l/l CA- | F | Previous affective disorder (MDD), anxiety disorder (Specific spider phobia) |
| l/l CA- | M | Previous affective disorder (MDD) Previous anxiety disorder (Panic attack) |
| l/l CA- | M | Previous NSSI, previous MDD |
| l/l CA- | M | Previous anxiety disorder (OCD & panic attacks) |
| s/s CA+ | F | Previous NSSI, affective disorder (MDD), anxiety disorder (panic disorder), alcohol abuse |
| s/s CA+ | F | Previous NSSI, affective disorder (MDD), anxiety disorder (panic disorder) |
| s/s CA+ | M | Previous behavioural disorder (CD), affective disorder (MDD), substance abuse |
| s/s CA+ | F | Previous NSSI |
| s/s CA+ | M | Previous NSSI, affective disorder (MDD) |
| s/s CA+ | F | Previous eating disorder |
| s/s CA+ | F | Previous affective disorder (MDD) |
| s/s CA+ | F | Previous affective disorder (MDD) |
| s/s CA- | M | Previous behavioural disorder (ADHD) |
| s/s CA- | M | Previous alcohol abuse |

Definitions: NSSI (non-suicidal self injury), MDD (Major depressive disorder), NOS (not otherwise-specified), HCI (high clinical index), CD (conduct disorder), ODD (oppositional defiant disorder), OCD (obsessive compulsive disorder), ADHD (attention deficit hyperactivity disorder). NSSI is reported although it is not a formal DSM-IV diagnosis.
