# Supplementary material for: 5-HTTLPR–environment interplay and its effects on neural reactivity in adolescents
Source: Neuroimage. 2012 Nov 15;63-248(3):1670–80. doi: 10.1016/j.neuroimage.2012.07.067 (PMC3480648; doi:10.1016/j.neuroimage.2012.07.067)
Supplement: Inline Supplementary Table S2 [file mmc2.docx]

Table S2. Correlation matrix of participant variables and amygdala reactivity.

| **Variable** | **Genotype** | **CA** | **RNLE14** | **RNLE17** | **SAI** | **MFQ** | **PH** | **LA Anger** | **LA Sad** | **LA Neutral** | **RA Anger** | **RA Sad** | **RA Neutral** |
| --- | --- | --- | --- | --- | --- | --- | --- | --- | --- | --- | --- | --- | --- |
| **Genotype Pearson correlation**  **Sig. (2-tailed)**  **N** | 1  67 | .044  .725  67 | -.259  .036  66 | -.021  .872  60 | .061  .628  65 | .033  .789  67 | .010  .938  67 | .288*  .018  67 | .152  .219  67 | .316**  .009  67 | .163  .188  67 | -.002  .988  67 | .266*  .030  67 |
| **CA Pearson correlation**  **Sig. (2-tailed)**  **N** | .044  .725  67 | 1  67 | .144  .249  66 | .210  .107  60 | .031  .806  65 | .241*  .050  67 | .310*  .011  67 | -.014  .911  67 | -.040  .748  67 | .070  .572  67 | .132  .285  67 | .027  .829  67 | .148  .233  67 |
| **RNLE14 Pearson correlation**  **Sig. (2-tailed)**  **N** | -.259*  .036  66 | .144  .249  66 | 1  66 | -.102  .438  60 | .039  .762  64 | -.019  .878  66 | .296*  .016  66 | -.097  .438  66 | -.087  .489  66 | -.164  .189  66 | -.137  .272  66 | -.082  .515  66 | -.093  .457  66 |
| **RNLE17 Pearson correlation**  **Sig. (2-tailed)**  **N** | -.021  .872  60 | .210  .107  60 | -.102  .438  60 | 1  60 | .297*  .024  58 | .175  .182  60 | -.160  .223  60 | .287*  .026  60 | .323  .012  60 | .333**  .009  60 | .219  .093  60 | .193  .140  60 | .263*  .042  60 |
| **SAI Pearson correlation**  **Sig. (2-tailed)**  **N** | .061  .628  65 | .031  .806  65 | .039  .762  64 | .297*  .024  58 | 1  65 | .420**  .000  65 | .078  .536  65 | .248*  .047  65 | .339**  .006  65 | .247*  .047  65 | .215  .085  65 | .260*  .037  65 | .178  .155  65 |
| **MFQ Pearson correlation**  **Sig. (2-tailed)**  **N** | .033  .789  67 | .241*  .050  67 | -.019  .878  66 | 175  .182  60 | .420**  .000  65 | 1  67 | .269*  .028  67 | .100  .419  67 | .197  .110  67 | .252*  .040  67 | .103  .406  67 | .144  .246  67 | .269*  .028  67 |
| **PH Pearson correlation**  **Sig. (2-tailed)**  **N** | .010  .938  67 | .310*  .011  67 | .296*  .016  66 | -.160  .223  60 | .078  .536  65 | .269*  .028  67 | 1  67 | -.200  .105  67 | -.216  .080  67 | -.089  .474  67 | -.143  .250  67 | -.126  .311  67 | .001  .995  67 |
| **LA Anger Pearson correlation**  **Sig. (2-tailed)**  **N** | .288*  .018  67 | -.014  .911  67 | -.097  .438  66 | .287*  .026  60 | .248*  .047  65 | .100  .419  67 | -.200  .105  67 | 1  67 | .718**  .000  67 | .683**  .000  67 | .844**  .000  67 | .584**  .000  67 | .625**  .000  67 |
| **LA Sad Pearson correlation**  **Sig. (2-tailed)**  **N** | .152  .219  67 | -.040  .748  67 | -.087  .489  66 | .323*  .012  60 | .339**  .006  65 | .197  .110  67 | -.216  .080  67 | .718**  .000  67 | 1  67 | .674**  .000  67 | .616**  .000  67 | .831**  .000  67 | .596**  .000  67 |
| **LA Neutral Pearson correlation**  **Sig. (2-tailed)**  **N** | .316**  .009  67 | .070  .572  67 | -.164  .189  66 | .333*  .009  60 | .247*  .047  65 | .252*  .040  67 | -.089  .474  67 | .683**  .000  67 | .674**  .000  67 | 1  67 | .543**  .000  67 | .558**  .000  67 | .820**  .000  67 |
| **RA Anger Pearson correlation**  **Sig. (2-tailed)**  **N** | .163  .188  67 | .132  .285  67 | -.137  .272  66 | .219  .093  60 | .215  .085  65 | .103  .406  67 | -.143  .250  67 | .844**  .000  67 | .616**  .000  67 | .543**  .000  67 | 1  67 | .713**  .000  67 | .684**  .000  67 |
| **RA Sad Pearson correlation**  **Sig. (2-tailed)**  **N** | -.002  .988  67 | .027  .829  67 | -.082  .515  66 | .193  .140  60 | .260*  .085  65 | .144  .246  67 | -.126  .311  67 | .584**  .000  67 | .831**  .000  67 | .558**  .000  67 | .713**  .000  67 | 1  67 | .675**  .000  67 |
| **RA Neutral Pearson correlation**  **Sig. (2-tailed)**  **N** | .266*  .030  67 | .148  .233  67 | -.093  .457  66 | .263*  .042  60 | .260*  .037  65 | .269*  .028  67 | .001  .995  67 | .625**  .000  67 | .596**  .000  67 | .820**  .000  67 | .684**  .000  67 | .675**  .000  67 | 1  67 |

Definitions: CA (childhood adversity), RNLE (recent negative life events) aged 14 or 17, PH (psychiatric history, SAI (Spielberger Anxiety Inventory), MFQ (Mood and Feelings Questionnaire), LA (left amygdala), RA (right amygdala).
