# Supplementary material for: 5-HTTLPR–environment interplay and its effects on neural reactivity in adolescents
Source: Neuroimage. 2012 Nov 15;63-248(3):1670–80. doi: 10.1016/j.neuroimage.2012.07.067 (PMC3480648; doi:10.1016/j.neuroimage.2012.07.067)
Supplement: Inline Supplementary Table S3 [file mmc3.docx]

**Table S3.** MANOVA 1 (No covariates)

| **ANALYSIS 1** |  |  |  |  |  |
| --- | --- | --- | --- | --- | --- |
| **Valence x Genotype x CA MANOVA**  **(No covariates)** |  |  |  |  |  |
|  |  |  |  |  |  |
| **Multivariate tests** |  |  |  |  |  |
| **Effect** |  |  |  |  |  |
| **Between Subjects** |  | **df** | **F** | **p** | **η_p_^2^** |
| Genotype* |  | 2,62 | 3.79 | 0.03 | 0.11 |
| CA |  |  | 1.38 | 0.26 | 0.04 |
| Genotype x CA |  |  | 0.20 | 0.82 | 0.01 |
|  |  |  |  |  |  |
| **Within Subjects** |  | **df** | **F** | **p** | **η_p_^2^** |
| Valence |  | 2,64 | 2.19 | 0.08 | 0.13 |
| Valence x Genotype |  |  | 1.88 | 0.13 | 0.11 |
| Valence x CA |  |  | 0.96 | 0.44 | 0.06 |
| Valence x Genotype x CA |  |  | 0.86 | 0.49 | 0.05 |
|  |  |  |  |  |  |
| **Univariate tests** |  |  |  |  |  |
| **Within Subjects** | **Hemisphere** | **df** | **F** | **p** | **η_p_^2^** |
| Valence* | L | 2,126 | 3.78 | 0.03 | 0.06 |
|  | R |  | 3.08 | 0.05 | 0.05 |
| Valence x Genotype* | L |  | 1.62 | 0.20 | 0.03 |
|  | R |  | 3.86 | 0.02 | 0.06 |
| Valence x CA | L |  | 0.67 | 0.51 | 0.01 |
|  | R |  | 0.73 | 0.49 | 0.01 |
| Valence x Genotype x CA | L |  | 0.25 | 0.78 | 0.00 |
|  | R |  | 0.31 | 0.74 | 0.01 |
|  |  |  |  |  |  |
| **Between Subjects** | **Hemisphere** | **df** | **F** | **p** | **η_p_^2^** |
| Genotype* | L | 1,63 | 5.53 | 0.02 | 0.08 |
|  | R |  | 1.47 | 0.23 | 0.02 |
| CA | L |  | 0.00 | 0.97 | 0.00 |
|  | R |  | 0.73 | 0.40 | 0.01 |
| Genotype x CA | L |  | 0.08 | 0.78 | 0.00 |
|  | R |  | 0.00 | 0.95 | 0.00 |

Abbreviations: CA (Childhood adversity), RNLE (Recent Negative Life Events), SAI (Spielberger Anxiety Inventory)

* Significant result at p < 0.05
