# Supplementary material for: 5-HTTLPR–environment interplay and its effects on neural reactivity in adolescents
Source: Neuroimage. 2012 Nov 15;63-248(3):1670–80. doi: 10.1016/j.neuroimage.2012.07.067 (PMC3480648; doi:10.1016/j.neuroimage.2012.07.067)
Supplement: Inline Supplementary Table S4 [file mmc4.docx]

**Table S4.** MANOVA 2 (with RNLE14, RNLE17, SAI and PH covariates)

| **ANALYSIS 2** |  |  |  |  |  |
| --- | --- | --- | --- | --- | --- |
| **Valence x Genotype x CA MANOVA**  **(with RNLE14, RNLE17, SAI, PH covariates)** |  |  |  |  |  |
|  |  |  |  |  |  |
| **Multivariate tests** |  |  |  |  |  |
| **Effect** |  |  |  |  |  |
| **Between Subjects** |  | **df** | **F** | **p** | **η_p_^2^** |
| Genotype* |  | 2,49 | 3.32 | 0.04 | 0.12 |
| CA |  |  | 1.14 | 0.32 | 0.04 |
| Genotype x CA |  |  | 0.26 | 0.77 | 0.01 |
| RNLE14 |  |  | 0.32 | 0.73 | 0.01 |
| RNLE17 |  |  | 2.73 | 0.08 | 0.10 |
| SAI |  |  | 0.92 | 0.40 | 0.04 |
| PH |  |  | 1.30 | 0.29 | 0.05 |
|  |  |  |  |  |  |
| **Within Subjects** |  | **df** | **F** | **p** | **η_p_^2^** |
| Valence |  | 4,48 | 0.70 | 0.60 | 0.06 |
| Valence x Genotype |  |  | 1.51 | 0.22 | 0.11 |
| Valence x CA |  |  | 1.60 | 0.19 | 0.12 |
| Valence x Genotype x CA |  |  | 0.60 | 0.66 | 0.05 |
| Valence x RNLE14 |  |  | 1.33 | 0.27 | 0.10 |
| Valence x RNLE17 |  |  | 0.46 | 0.77 | 0.37 |
| Valence x SAI |  |  | 0.70 | 0.60 | 0.06 |
| Valence x PH |  |  | 0.73 | 0.58 | 0.06 |
|  |  |  |  |  |  |
| **Univariate tests** |  |  |  |  |  |
| **Within Subjects** | **Hemisphere** | **df** | **F** | **p** | **η_p_^2^** |
| Valence | L | 2,100 | 1.27 | 0.29 | 0.03 |
|  | R |  | 0.74 | 0.48 | 0.02 |
| Valence x Genotype | L |  | 0.53 | 0.59 | 0.01 |
|  | R |  | 2.60 | 0.08 | 0.05 |
| Valence x CA | L |  | 0.26 | 0.80 | 0.01 |
|  | R |  | 0.61 | 0.53 | 0.01 |
| Valence x Genotype x CA | L |  | 0.07 | 0.93 | 0.00 |
|  | R |  | 0.14 | 0.87 | 0.00 |
| Valence x RNLE14 | L |  | 0.29 | 0.75 | 0.01 |
|  | R |  | 0.45 | 0.64 | 0.01 |
| Valence x RNLE17 | L |  | 0.20 | 0.82 | 0.00 |
|  | R |  | 0.75 | 0.48 | 0.02 |
| Valence x SAI | L |  | 1.02 | 0.37 | 0.02 |
|  | R |  | 0.96 | 0.39 | 0.02 |
| Valence x PH | L |  | 0.70 | 0.50 | 0.01 |
|  | R |  | 1.15 | 0.32 | 0.02 |
|  |  |  |  |  |  |
| **Between Subjects** | **Hemisphere** | **df** | **F** | **p** | **η_p_^2^** |
| Genotype* | L | 1,50 | 3.97 | 0.05 | 0.07 |
|  | R |  | 0.54 | 0.47 | 0.01 |
| CA | L |  | 0.06 | 0.84 | 0.00 |
|  | R |  | 1.01 | 0.31 | 0.02 |
| Genotype x CA | L |  | 0.04 | 0.84 | 0.00 |
|  | R |  | 0.31 | 0.58 | 0.01 |
| RNLE14 | L |  | 0.00 | 1.00 | 0.00 |
|  | R |  | 0.20 | 0.66 | 0.00 |
| RNLE17* | L |  | 4.08 | 0.05 | 0.08 |
|  | R |  | 0.99 | 0.30 | 0.02 |
| SAI | L |  | 1.87 | 0.18 | 0.04 |
|  | R |  | 1.45 | 0.23 | 0.03 |
| PH | L |  | 2.33 | 0.13 | 0.05 |
|  | R |  | 1.03 | 0.32 | 0.02 |

Abbreviations: CA (Childhood adversity), RNLE (Recent Negative Life Events), SAI (Spielberger Anxiety Inventory), PH (Psychiatric history).

* Significant result at p < 0.05
