# Supplementary material for: 5-HTTLPR–environment interplay and its effects on neural reactivity in adolescents
Source: Neuroimage. 2012 Nov 15;63-248(3):1670–80. doi: 10.1016/j.neuroimage.2012.07.067 (PMC3480648; doi:10.1016/j.neuroimage.2012.07.067)
Supplement: Inline Supplementary Table S5 [file mmc5.docx]

**Table S5.** Whole brain effects (no covariates): threshold at p < 0.01, 30 voxel cluster threshold.

| **Comparison** | **Region** | **Cluster size (k_E_)** | **p (FWE-corr)** | **F** | **Z** | **X** | **Y** | **Z** |
| --- | --- | --- | --- | --- | --- | --- | --- | --- |
| *Main effect of genotype* | *Cuneus (BA17) | 992 | .008 | 28.05 | 4.97 | -6 | -98 | 0 |
|  |  |  | .142 | 20.71 | 4.28 | -28 | -66 | -16 |
|  |  |  | .276 | 18.84 | 4.07 | -16 | -98 | 6 |
|  | Cerebellum | 185 | .291 | 18.68 | 4.06 | 32 | -50 | -26 |
|  | Inferior occipital gyrus (BA18) | 58 | .598 | 16.25 | 3.77 | 36 | -76 | -6 |
|  | Inferior temporal gyrus (BA37) | 37 | .724 | 15.39 | 3.67 | -36 | -70 | -2 |
|  | Cerebellum | 71 | .763 | 15.11 | 3.63 | 44 | -62 | -28 |
|  |  |  |  |  |  |  |  |  |
| *Main effect of CA* | *Cuneus (BA19) | 128 | .012 | 27.04 | 4.89 | 30 | -92 | 26 |
|  | Cuneus (BA19) | 271 | .054 | 23.22 | 4.53 | -22 | -96 | 28 |
|  |  |  | .068 | 22.64 | 4.47 | -20 | -102 | 18 |
|  |  |  | .343 | 18.19 | 4.00 | -26 | -88 | 12 |
|  |  |  |  |  |  |  |  |  |
| *Genotype x CA interaction* | *Lingual gyrus (BA17) | 68 | .023 | 25.35 | 4.73 | -20 | -84 | -4 |

* Significant at p < 0.05 Family-wise error corrected
