# Supplementary material for: 5-HTTLPR–environment interplay and its effects on neural reactivity in adolescents
Source: Neuroimage. 2012 Nov 15;63-248(3):1670–80. doi: 10.1016/j.neuroimage.2012.07.067 (PMC3480648; doi:10.1016/j.neuroimage.2012.07.067)
Supplement: Inline Supplementary Table S6 [file mmc6.docx]

**Table S6**. Whole-brain effects of RNLE14, RNLE17, SAI and PH covariates.

| **Comparison** | **Region** | **Cluster size (k_E_)** | **p (FWE-corr)** | **T** | **Z** | **X** | **Y** | **Z** |
| --- | --- | --- | --- | --- | --- | --- | --- | --- |
|  |  |  |  |  |  |  |  |  |
| *Positive effect of RNLE14* |  | 75 | .087 | 4.51 | 4.36 | -30 | -68 | 52 |
|  |  |  |  |  |  |  |  |  |
| *Negative effect of RNLE14* |  | 48 | .288 | 4.12 | 4.01 | 38 | -82 | -14 |
|  |  | 89 | .320 | 4.08 | 3.97 | -38 | -50 | -4 |
|  |  | 69 | .621 | 3.78 | 3.69 | -24 | -94 | 28 |
|  |  | 40 | .736 | 3.67 | 3.59 | 28 | -90 | 2 |
|  |  |  | .919 | 3.43 | 3.37 | 30 | -88 | -6 |
|  |  |  |  |  |  |  |  |  |
| *Positive effect of RNLE17* | * Precentral gyrus (BA6) | 226 | <.001 | 6.91 | 6.45 | 48 | -2 | 38 |
|  | * Precentral gyrus (BA6) | 182 | .007 | 5.19 | 4.98 | -50 | -4 | 50 |
|  |  |  | .687 | 3.72 | 3.63 | -38 | 0 | 38 |
|  | * Inferior frontal gyrus (BA45) | 177 | .041 | 4.72 | 4.56 | -54 | 24 | 20 |
|  |  |  | .570 | 3.82 | 3.73 | -52 | 28 | 28 |
|  | Middle frontal gyrus (BA46) | 74 | .098 | 4.47 | 4.33 | 50 | 36 | 32 |
|  | Fusiform gyrus (BA37) | 139 | .144 | 4.35 | 4.22 | 48 | -60 | -16 |
|  |  |  | .271 | 4.14 | 4.03 | 48 | -80 | -8 |
|  |  |  | .376 | 4.02 | 3.91 | 48 | -74 | -16 |
|  | Fusiform gyrus (BA37) | 90 | .152 | 4.34 | 4.21 | 44 | -42 | -24 |
|  | Fusiform gyrus (BA37) | 74 | .154 | 4.33 | 4.20 | -48 | -46 | -18 |
|  | Inferior parietal lobule (BA40) | 83 | .195 | 4.25 | 4.13 | -40 | -58 | 56 |
|  |  |  | .969 | 3.31 | 3.25 | -40 | -50 | 42 |
|  | Lingual gyrus (BA18) | 58 | .201 | 4.24 | 4.12 | 14 | -96 | -10 |
|  | Inferior parietal lobule (BA40) | 33 | .354 | 4.04 | 3.93 | 44 | -58 | 58 |
|  | Inferior temporal gyrus (BA20) | 186 | .362 | 4.03 | 3.93 | -58 | -26 | -22 |
|  |  |  | .579 | 3.82 | 3.73 | -36 | -18 | -28 |
|  |  |  | .586 | 3.81 | 3.72 | -48 | -26 | -18 |
|  | Precuneus (BA39) | 116 | .401 | 3.99 | 3.89 | 36 | -68 | 34 |
|  |  |  | .790 | 3.61 | 3.53 | 30 | -66 | 22 |
|  | Superior temp. gyrus (BA22) | 68 | .454 | 3.93 | 3.89 | -64 | -40 | 12 |
|  | Superior temp. gyrus (BA22) | 94 | .464 | 3.93 | 3.83 | 60 | -50 | 14 |
|  | Caudate | 92 | .563 | 3.83 | 3.74 | -12 | 10 | 12 |
|  | Superior frontal gyrus (BA10) | 35 | .667 | 3.73 | 3.65 | 14 | 58 | 24 |
|  |  |  |  |  |  |  |  |  |
| *Negative effect of RNLE17* | Insula (BA13) | 61 | .608 | 3.79 | 3.70 | 40 | 12 | 14 |
|  |  |  |  |  |  |  |  |  |
| *Positive effect of SAI* | *Precentral gyrus (BA6) | 214 | .031 | 4.80 | 4.63 | -38 | -2 | 30 |
|  | Middle occipital gyrus (BA37) | 96 | .105 | 4.45 | 4.31 | -40 | -76 | 0 |
|  | Middle occipital gyrus (BA18) | 85 | .120 | 4.41 | 4.28 | -32 | -96 | -6 |
|  | Middle frontal gyrus (BA6) | 74 | .130 | 4.38 | 4.25 | 12 | -2 | 56 |
|  | Middle occipital gyrus (BA18) | 61 | .214 | 4.22 | 4.10 | 16 | -90 | 14 |
|  | Middle occipital gyrus (BA18) | 58 | .605 | 3.79 | 3.70 | -12 | -96 | 12 |
|  | Cerebellum | 50 | .648 | 3.75 | 3.67 | 24 | -42 | -26 |
|  | Cerebellum | 30 | .865 | 3.52 | 3.45 | -38 | -22 | 64 |
|  |  |  |  |  |  |  |  |  |
| *Negative effect of SAI* | No significant clusters |  |  |  |  |  |  |  |
|  |  |  |  |  |  |  |  |  |
| *Positive effect of PH.* | No significant clusters |  |  |  |  |  |  |  |
|  |  |  |  |  |  |  |  |  |
| *Negative effect of PH* | Lingual gyrus (BA18) | 54 | .233 | 4.19 | 4.08 | -18 | -84 | -2 |
|  | Cingulate gyrus (BA24) | 103 | .289 | 4.12 | 4.01 | 10 | -24 | 38 |
|  |  |  | .975 | 3.29 | 3.23 | 14 | -10 | 38 |
|  | Inferior temporal gyrus (BA19) | 42 | .580 | 3.82 | 3.73 | 48 | -60 | -6 |
|  | Cerebellum | 43 | .674 | 3.73 | 3.64 | 34 | -58 | -24 |
|  | Inferior temporal gyrus (BA37) | 82 | .678 | 3.72 | 3.64 | -46 | -72 | -4 |
|  | Cuneus (BA18) | 66 | .727 | 3.68 | 3.60 | -2 | -92 | 16 |
|  | Cerebellum | 59 | .731 | 3.67 | 3.59 | -26 | -72 | -22 |
|  |  | 31 | .753 | 3.65 | 3.57 | -12 | -86 | -18 |
|  |  | 31 | .815 | 3.58 | 3.51 | 28 | 16 | 62 |
|  | Middle temporal gyrus (BA39) | 54 | .642 | 3.76 | 3.67 | 46 | -76 | 20 |
|  |  |  |  |  |  |  |  |  |

* Significant at p < 0.05 Family-wise error corrected for multiple comparisons. RNLE = Recent Negative Life Events, SAI = Spielberger State Anxiety Inventory, PH = Psychiatric History.
