# Supplementary material for: 5-HTTLPR–environment interplay and its effects on neural reactivity in adolescents
Source: Neuroimage. 2012 Nov 15;63-248(3):1670–80. doi: 10.1016/j.neuroimage.2012.07.067 (PMC3480648; doi:10.1016/j.neuroimage.2012.07.067)
Supplement: Supplementary file 7 — Supplementary materials [file mmc7.docx]

**Supplemental Results: 5-HTTLPR-environment interplay and its effects on neural reactivity in adolescents.**

**Supplemental fMRI results**

Following the primary analyses described in the main text we ran a number of supplementary analyses.

*Effects of psychiatric history (PH) upon amygdala activation.*

We next investigated the role of psychiatric history (PH) upon amygdala activation. Twenty one individuals received a previous diagnosis through K-SADS interview when assessed as part of the ROOTS study (see table S1); 15 of the individuals were from the adversity group, and there was an even split with genotype. At the time of scanning, PH+ scored significantly higher on the MFQ than PH- participants (F_1,63_ = 4.79, p = .032, ƞ_p_^2^ = 0.071), with no difference in IQ, RNLE14, RNLE17, or SAI scores. In terms of amygdala reactivity, there was no difference between the PH+ and PH- groups.

*Effect of triallelic classification upon amygdala reactivity.*

We investigated the effect of grouping participants according to the triallelic classification as described in previous reports (e.g. (Lau et al., 2009)). However it should be noted that this analysis was not planned *a priori* and so is consequently underpowered. In this analysis we ran analysis (2) as described in the main paper with SAI, RNLE and PH as covariates, but replacing the biallelic genotype classification with the triallelic classification. When this analysis was run there were no significant main effects or interactions.

*Confirmatory genotype by CA random effects analysis*

Following a reviewer suggestion that the Genotype x CA x Valence second-level analysis reported in the main text did not fully model any within-subjects effects, we ran a confirmatory second-level analysis to ascertain that our whole-brain results were not biased by any un-modelled within-subject variance. In this analysis we performed a first-level fixed effects analysis to create an all-faces vs. fixation contrast image. A 2 (genotype) x 2 (CA) second-level random effects analysis was then run.

In this analysis we were able to confirm the main effect of genotype in the left cuneus cluster identified in the original whole-brain analysis (see Figure S3) and an identical genotype x CA interaction in the left lingual gyrus cluster identified in the original whole-brain analysis (see Figure S4). However, due to the reduced degrees of freedom in this 2 x 2 model (compared to the original 2 x 2 x 3) model mean reduced power in this analysis as reflected in the *F* and *z*-score for these results. For the lingual gyrus cluster (-20,-84, -4) in the genotype x CA interaction 2 x 2 x 3 model F = 38.71, z = 5.76, df = 1, 159). For the lingual gyrus cluster (-20,-84, -4) in the genotype x CA interaction 2 x 2 model F = 15.97, z = 3.53, df = 1,51.

**Supplementary figure legends**

**Figure S1.** Additive effects of 5-HTTLPR genotype and recent negative life events (RNLE) upon amygdala reactivity. This figure was generated by performing a median split on RNLE to form two groups that either had or had not experienced RNLE.

**Figure S2.** Glass-brain rendering of covariate effects: recent negative life events aged 14 (RNLE14) and aged 17 (RNLE17); Spielberger state anxiety inventory scores (SAI) and psychiatric history (PH). Note RNLE17 and SAI show a significant positive effect on brain reactivity, in contrast RNLE14 and PH shows a significant negative effect on brain reactivity.

**Figure S3.** Supplemental confirmatory results of all faces vs. fixation contrast (RNLE14, RNLE17, SAI, and PH covaried): main effect of genotype. Activation in left cuneus (BA17) [Montreal Neurological Institute coordinates -6,-98,-2] overlaid on axial section and thresholded at p < 0.01. Intensity bar represents F score.

**Figure S4.** Supplemental confirmatory results of all faces vs. fixation contrast (RNLE14, RNLE17, SAI, and PH covaried): genotype by CA interaction. Activation present in left lingual gyrus (BA19) [Montreal Neurological Institute coordinates -20,-84,-4] overlaid on axial section and thresholded at p < 0.01. Intensity bar represents F score.

**Supplemental References**

Lau, J.Y., Goldman, D., Buzas, B., Fromm, S.J., Guyer, A.E., Hodgkinson, C., Monk, C.S., Nelson, E.E., Shen, P.H., Pine, D.S., Ernst, M., 2009. Amygdala function and 5-HTT gene variants in adolescent anxiety and major depressive disorder. Biol Psychiatry 65, 349-355.
